# Ozone pollution reduction partially offsets the negative impact of climate change mitigation efforts on global hunger

**DOI:** 10.1038/s43016-026-01322-3

**Published:** 2026-03-16

**Authors:** Shujuan Xia, Tomoko Hasegawa, Thanapat Jansakoo, Daniel Mason-D’Croz, Kazuaki Tsuchiya, Shinichiro Fujimori, Maksym Chepeliev, Marta Kozicka, Abhijeet Mishra, Willem-Jan van Zeist, Xin Zhao, Thijs de Lange, Thais Diniz Oliveira, Jonathan C. Doelman, Matthew Gibson, Petr Havlík, Mario Herrero, Ipsita Kumar, Yuki Ochi, Timothy B. Sulser, Marina Sundiang, Kiyoshi Takahashi, Jun’ya Takakura, Keith Wiebe

**Affiliations:** 1 Social Systems Division, National Institute for Environmental Studies, Tsukuba, Japan; 2 Research Organization of Science and Technology, Ritsumeikan University, Kusatsu, Japan; 3 Department of Environmental Engineering, Kyoto University, Kyoto, Japan; 4 Department of Environment for Sustainability, Faculty of Environment, Kasetsart University, Bangkok, Thailand; 5 Department of Global Development, College of Agriculture and Life Sciences, Cornell University, Ithaca, NY USA; 6 Cornell Atkinson Center for Sustainability, Cornell University, Ithaca, NY USA; 7 Agricultural Economics and Rural Policy Group, Wageningen University and Research, Wageningen, the Netherlands; 8 International Institute for Applied Systems Analysis, Laxenburg, Austria; 9 Purdue University, West Lafayette, IN USA; 10 International Food Policy Research Institute, Washington DC, USA; 11 Potsdam Institute for Climate Impact Research, Potsdam, Germany; 12 Wageningen Social & Economic Research, Wageningen University and Research, The Hague, the Netherlands; 13 Joint Global Change Research Institute, Pacific Northwest National Laboratory, College Park, MD USA; 14 Center for Global Sustainability, School of Public Policy, University of Maryland, College Park, MD USA; 15 PBL Netherlands Environmental Assessment Agency, The Hague, the Netherlands; 16 E-Konzal Co. Ltd, Osaka, Japan; 17 Present Address: Atmosphere and Ocean Research Institute, The University of Tokyo, Chiba, Japan

**Keywords:** Climate-change mitigation, Agriculture

## Abstract

Studies warning of the potential negative effects of climate mitigation on food security through the competing use of land for bioenergy and afforestation have overlooked the impact of reduced ozone and its potential enhancement of crop yields. Here we use six global agro-economic models to compare the impacts of climate change with climate mitigation policy and ozone reduction on agriculture. We find that ozone reduction could reduce the negative impact of a 1.5 °C-consistent climate change mitigation policy on global hunger by 15% in 2050. Sub-Saharan Africa and India, where hunger is most severe, account for 56% of this global reduction. Our findings indicate that the negative effects of climate mitigation on global hunger could be partially offset by the ozone reduction impact.

## Abstract (web summary)

A multi-model intercomparison using six global agro-economic models explores how ozone reductions arising from climate change mitigation could impact crop yields. Yield changes are projected to 2050, linking reduced ozone to food security and the alleviation of hunger.

## Main

Ensuring food security and eradicating global hunger for future populations are major global challenges and key components of the United Nations’ Sustainable Development Goals, particularly SDG2: Zero Hunger. Achieving this goal requires a deep understanding of the factors contributing to hunger and food insecurity. Climate change poses substantial threats to food security by altering crop yields, disrupting agricultural production and exacerbating environmental, socio-economic and infrastructural vulnerabilities in food systems, including crops, livestock, aquaculture and fisheries^1,2^. Reduced crop yields can lead to higher food prices and reduced food availability, impacting food security, particularly for vulnerable populations who may already be experiencing food scarcity^3,4^. Climate change mitigation policies to reduce greenhouse gas (GHG) emissions are indispensable for safeguarding long-term food security.

The Paris Agreement, adopted in 2015, calls on countries to limit the rise in global average temperature to below 2 °C above pre-industrial levels, with efforts to keep it under 1.5 °C, by the end of the century^5^. While this ambitious goal for reducing GHG emissions aims to lessen the negative effects of global warming, it may require increased bioenergy production and afforestation, raising concerns about food security^6–8^. For instance, according to a single global agro-economic model (AIM-Hub) estimate, competition for land driven by bioenergy production and the costs of these mitigation efforts could lead to higher food prices, putting more people at risk of hunger by 2050^6^. Meanwhile, mitigation measures to reduce GHG emissions, such as cutting fossil fuel use and promoting renewable energy, often lower co-emitted air pollutants such as methane (CH_4_), nitrogen oxides (NO_*x*_) and volatile organic compounds (VOCs)^9–12^. The agricultural sector is a notable source of CH_4_ (ref. ^13^) and NO_*x*_ emissions^14^, and agricultural mitigation measures, such as improved soil carbon management and the use of methane-inhibiting feed additives, can help reduce these emissions^15,16^.

**Fig. 1:**
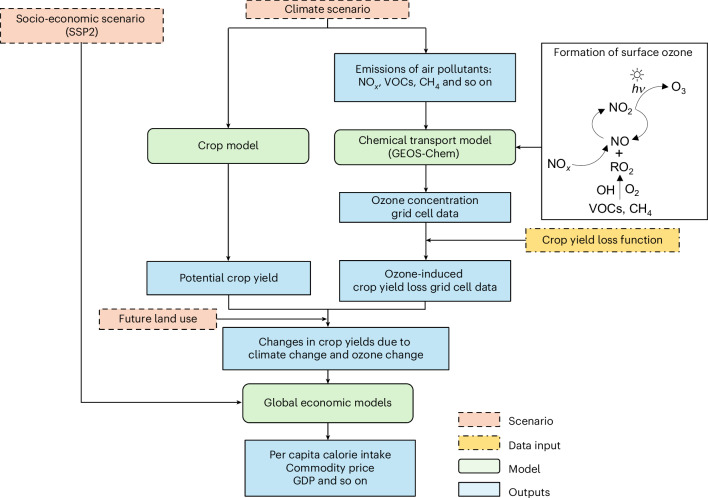
Modelling framework for the scenario analysis. The column on the right illustrates a simplified process of surface ozone formation^18^. *hv* denotes sunlight (photon energy) driving photochemical reactions in surface ozone formation. The global economic models mentioned refer to the six global agricultural economic models used in this study: AIM-Hub, ENVISAGE, GCAM, GLOBIOM, IMPACT and MAGNET. GDP, gross domestic product.

Surface ozone (hereafter, ozone) is primarily formed through photochemical reactions involving the oxidation of CH_4_ and other non-methane VOCs, in the presence of NO_*x*_ (Fig. 1)^17,18^. Elevated ozone concentrations, resulting from increased emissions of these air pollutants, reduce crop production^19–23^, cause economic damage (for example, US$34–45 billion in crop production losses)^24,25^ and increase premature mortality^26,27^. Reducing the emission of these pollutants can therefore lower ozone concentrations and mitigate these adverse impacts^9–11,28^. As an accompanying effect of GHG emission mitigation, ozone reduction is expected to offset a portion of the effects of rising temperatures on crop yields, particularly in low- and middle-income countries^29^. Additionally, ozone reduction from stringent mitigation measures could limit crop yield losses to approximately 10% by 2050, especially in Asia, compared with higher losses without this effect, according to a single integrated assessment model (IMAGE)^30^.

Multi-model intercomparisons can assess the uncertainties inherent in complex issues such as the impacts of climate change and air pollution on food security^31^. Recent multi-model assessments suggest that stringent mitigation efforts could have more severe negative impacts on global hunger than unmitigated climate change, as they may increase food prices and reduce calorie availability^32–34^. However, these studies have not accounted for the ozone reduction impacts that accompany mitigation efforts, which raises key questions: if these impacts are considered, how might they affect estimates of climate-policy-related impacts on food prices and food security? To what extent could this accompanying effect reduce negative mitigation impacts, suggesting that previous studies may have overestimated the adverse effects of climate mitigation on food security? To date, no studies have assessed this potential in a multi-model intercomparison framework, leaving a critical gap in our understanding.

To address the above questions, we present a multi-model intercomparison using six global agro-economic models—AIM-Hub^35^, ENVISAGE^36^, GCAM^37^, GLOBIOM^38^, IMPACT^39^ and MAGNET^40^—that simulate the interplay between crop production, trade, prices and food availability. Building on modelling efforts that support the second EAT-Lancet Commission report^41,42^, we first considered a baseline scenario with no additional changes in climate or ozone concentrations from the current levels but including currently implemented national policies (NPis) with a cut-off year of 2020 (Table 1). Five additional scenarios were developed to investigate the individual and combined effects of climate change, mitigation efforts and ozone reduction, each representing different combinations of these three factors (Table 1). Specifically, we based our analysis on the SSP–RCP scenario framework developed for the Coupled Model Intercomparison Project Phase 6, which combines Shared Socio-economic Pathways (SSPs) with Representative Concentration Pathways (RCPs)^43^. We used SSP2—representing a “middle-of-the-road” socio-economic development trajectory^44^—combined with two different radiative forcing levels: RCP2.6 (strong mitigation aimed at limiting warming to below 1.5 °C) and RCP7.0 (a high-emissions pathway with limited mitigation). These combinations, SSP2-2.6 (mitigation scenario) and SSP2-7.0 (business-as-usual scenario), allowed us to assess a range of plausible future climate and policy environments.

**Table 1:** Scenarios used in this study

Scenario	Name	Climate mitigation policy	Climate conditions	Ozone changes	Impacts analysed
Baseline	BAU_NoCC	No policy	NoCC	No changes	
SSP2-2.6	BAU_MITI	RCP2.6	RCP7.0	No changes	M
	BAU_MITI_RCP26	RCP2.6	RCP2.6	No changes	C + M
	OZBAU_MITI_RCP26	RCP2.6	RCP2.6	RCP2.6	C + M + O
SSP2-7.0	BAU	No policy	RCP7.0	No changes	C
	OZBAU	No policy	RCP7.0	RCP7.0	C + O

‘No policy’ indicates no emission constraints and no mitigation policy. NoCC, no climate change, assuming the current climate and air pollution (ozone concentration) conditions persist; C, impact of climate change; M, impact of stringent mitigation (without accounting for the accompanying ozone reduction); O, impact of the accompanying ozone concentration change.

Crop yield changes due to climate change are based on projections from the Global Gridded Crop Model Intercomparison^45^ of the Agricultural Model Intercomparison and Improvement Project (AgMIP). Productivity shocks on livestock^46^ and agricultural labour^47^ resulting from heat exposure are also incorporated on the basis of previous projections. Mitigation policies targeting SSP2-2.6 are implemented through a global carbon tax applied across agricultural, land use and other sectors. Stringent mitigation efforts in SSP2-2.6 result in lower ozone concentrations than SSP2-7.0, where mitigation efforts are minimal under NPi assumptions, and air quality improvements rely primarily on technological advancements and economic development. The resulting change in crop yields due to ozone reduction from the baseline is projected using crop-specific exposure–response relationships^21,48^. Each model reports food-security-relevant results such as crop production and mean per capita calorie consumption. Using these outputs—including minimum energy requirements and the coefficient of variation of dietary energy consumption within countries—we estimate the population at risk of hunger by 2050, following the methods of previous studies^32–34^. See Methods for more details about the overall research framework.

## Results

### Projected hunger risk in the baseline scenario

**Fig. 2:**
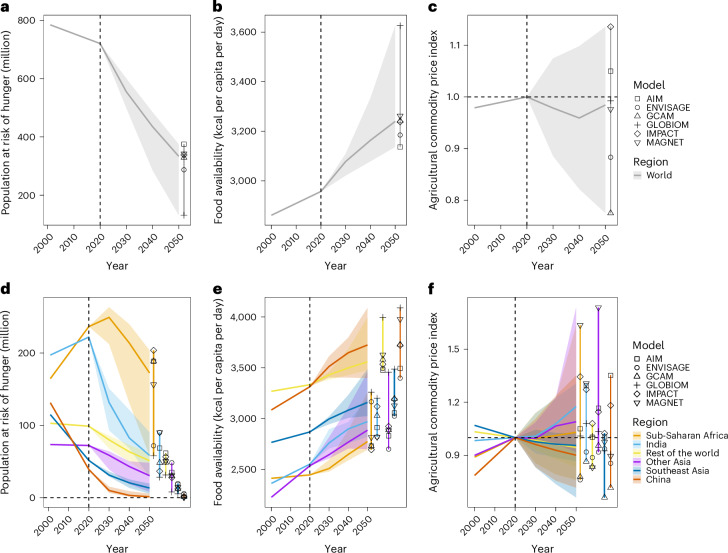
Projected global and regional food security in the baseline scenario. **a**–**f**, Global (**a**–**c**) and regional (**d**–**f**) trends in the population at risk of hunger (**a**,**d**), food availability (**b**,**e**) and agricultural commodity prices (**c**,**f**). The solid lines represent the median values across multiple models. In **c** and **f**, the agricultural commodity price in 2020 is set at 1 (that is, the unit of this price in 2020 equals 1), as indicated by horizontal dashed lines. Agricultural commodities include both food products and feed crops grown for livestock consumption. The vertical dashed lines indicate the year before the first year of the projection. The shaded areas indicate the ranges across the model estimates, and the markers within the vertical bars indicate results from each global agricultural economic model in 2050: AIM-Hub, ENVISAGE, GCAM, GLOBIOM, IMPACT and MAGNET.

Globally, hunger risk has recently increased, but the mean projected hunger risk is expected to decline by 2050 (Fig. 2a) in the baseline scenario, due to increased food availability (Fig. 2b) resulting primarily from income growth in low- and middle-income countries (Supplementary Fig. 1). Food availability is projected to increase from 2,960 kcal in 2020 to a multi-model median of 3,240 kcal (3,140–3,630 kcal; this range indicates inter-model variability hereinafter) per capita per day by 2050. Consequently, the global population at risk of hunger will decrease by 386 million (345–589 million) in 2050 compared with 2020, reducing this number to 334 million (131–375 million; 1–4% of the total population). The model ensemble projects modest changes in agricultural commodity price between 2020 and 2050, ranging from 0.88 (–12%) to 1.14 (+14%) relative to 2020 levels (Fig. 2c). These trends are consistent with earlier studies^32–34^.

Regionally, the model median projection shows agricultural food prices increasing in sub-Saharan Africa, India and other Asian countries while decreasing in other regions over time (Fig. 2f). However, food availability is projected to increase steadily over time across all regions (Fig. 2e). As a result, the population at risk of hunger is projected to continuously decrease by 2050 in most regions, except for sub-Saharan Africa and India (Fig. 2d). In sub-Saharan Africa, hunger risks are projected to increase until 2030, despite rising food availability, primarily due to population growth (Supplementary Fig. 2). Currently, sub-Saharan Africa has the largest share of the global hunger risk at ~33% (237 million; 21% of the total population), followed by India at ~31% (222 million; 16% of the total population). By 2050, India is projected to experience the largest decline in hunger, with the affected population decreasing to nearly 58 million (28–90 million; 2–5% of the total population), followed by sub-Saharan Africa with a decrease to 172 million (58–204 million; 3–11% of the total population). These declines are largely attributed to increased calorie intake driven by rapid income growth (Supplementary Fig. 1). However, India and sub-Saharan Africa will still account for the largest shares of global hunger by 2050, with a decrease to 18% (11–31%) and a rise to 53% (25–60%), respectively.

### Trade-offs of climate mitigation and accompanying ozone reduction impacts on food security

**Fig. 3:**
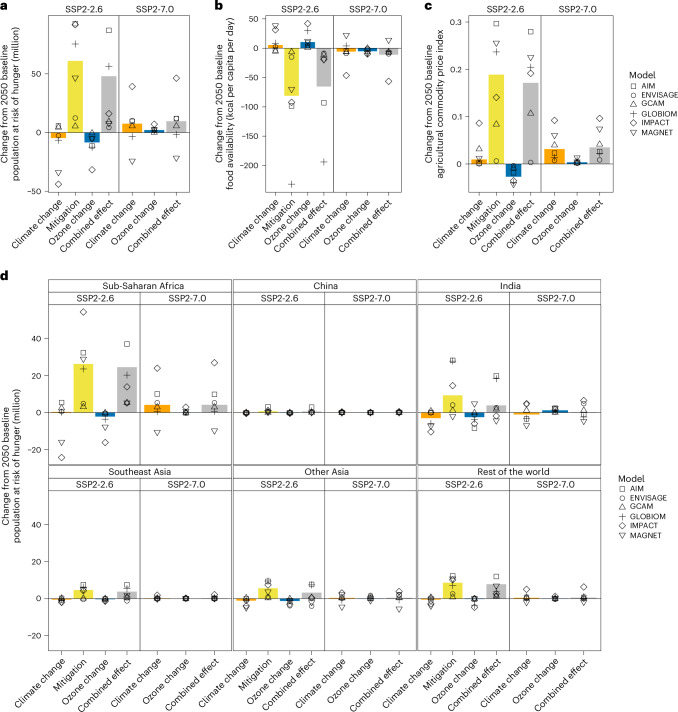
Food security under climate change and mitigation scenarios. **a**–**c**, Changes in the global population at risk of hunger (**a**), food availability (**b**) and agricultural food commodity prices (**c**) from the baseline scenario in 2050, resulting from each individual factor and their combined effects. **d**, Regional changes in the population at risk of hunger because of each factor and the combined effect of all three factors. The bars indicate the multi-model median, whereas the symbols represent the results from individual global agricultural economic models: AIM-Hub, ENVISAGE, GCAM, GLOBIOM, IMPACT and MAGNET. Ozone change serves as an accompanying effect of changes in air pollutant emissions in each scenario, whereas the mitigation effect refers to the negative impacts of these efforts without accounting for the accompanying ozone reduction. The decomposition of these three factors is described in Methods and in Supplementary Table 1.

Global warming in the SSP2-7.0 scenario tends to progressively reduce food availability and increase hunger risks compared with the baseline scenario (Supplementary Fig. 3), raising the global population at risk of hunger by 7.5 million (5–39 million in most models) by 2050 (Fig. 3a). Although NO_*x*_ and VOC emissions decrease relative to the baseline scenario, CH_4_ emissions increase over time (Supplementary Fig. 4), leading to higher ozone concentrations. As a result, elevated ozone levels reduce crop yields (Supplementary Fig. 5), which in turn increase food prices and decrease food availability, pushing an additional 2 million (0.03–7 million) people into hunger. In total, 9.5 million more people are projected to face hunger by 2050 (Fig. 3a–c). In SSP2-2.6, a carbon tax is implemented under climate change mitigation to achieve the 1.5 °C climate goal. This reduces warming, which boosts crop yields and food availability, lowering the number of people at risk of hunger by 5 million (3–44 million in most models) compared with the baseline. However, mitigation measures alone increase production costs, land rent and agricultural commodity producer prices to more than sixfold higher than the increase caused by warming in SSP2-7.0, resulting in a decrease in calorie intake of 81 kcal (7–232 kcal) per capita per day and an increase in hunger risk of 61 million (5–93 million) people (Fig. 3a–c and Supplementary Fig. 6), consistent with earlier findings^23–25^. Notably, mitigation measures in SSP2-2.6 reduce co-emitted air pollutants, particularly CH_4_, NO_*x*_ and VOCs (Supplementary Fig. 4), which are ozone precursors, leading to lower ozone levels. These ozone reductions lead to increased crop yields (Supplementary Fig. 5). The resulting yield gains reduce food prices, thereby enhancing food availability and mitigating the adverse effects of mitigation-related costs on hunger risk. By 2050, ozone reduction is projected to reduce the number of people at risk of hunger by 8.4 million (0.5–32 million), offsetting approximately 15% of the negative effects associated with mitigation.

Regionally, similar to global trends, hunger risks increase due to reduced crop yields, decreased food availability and rising food prices (Supplementary Fig. 7). The largest changes occur in sub-Saharan Africa and India in both scenarios. In SSP2-7.0, warming could increase hunger risks for a multi-model median of 4.1 million people (0.3% of the total population) in sub-Saharan Africa and between 0.04 and 0.4 million in other regions, compared with the baseline scenario (Fig. 3d). India is an exception, where warming does not reduce calorie intake (Supplementary Fig. 8) but results in 0.9 million fewer people at risk of hunger. Elevated ozone levels driven by increased CH_4_ emissions raise hunger risk across all regions, with the largest impact occurring in India, where the risk increases by a multi-model median of 1.2 million people (0.07% of the total population). In SSP2-2.6, hunger risks intensify (Supplementary Fig. 9), with stringent mitigation resulting in a multi-model median increase of 26 and 6.3 million people (1% and 0.4% of the total population) at risk of hunger in sub-Saharan Africa and India, respectively. However, ozone reduction from mitigation decreases hunger risk by 2.2 million in sub-Saharan Africa and 2.5 million in India, offsetting 8% and 39% of the negative impacts of mitigation in these regions, respectively. Together, they account for the majority of the global offsetting effects, with sub-Saharan Africa accounting for 26% and India for 30% of the total.

**Fig. 4:**
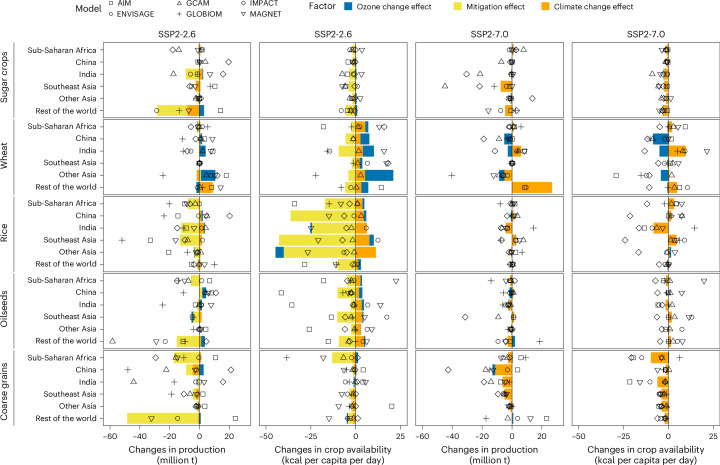
Changes in crop production and crop-based food availability owing to climate change and mitigation. The bars represent the median levels across models, indicating the change from the baseline scenario without additional climate change and ozone pollution in 2050. The symbols represent the combined effects of the three factors for each global agricultural economic model: AIM-Hub, ENVISAGE, GCAM, GLOBIOM, IMPACT and MAGNET.

The notable reduction in hunger risk in India due to ozone reduction is primarily driven by substantial increases in wheat production, boosting calorie intake (Fig. 4 and Supplementary Fig. 10). Similar trends can be observed in other key wheat-producing regions such as China and other Asian countries (Supplementary Fig. 11). Although the absolute reduction in hunger risk is relatively small in these regions, ozone reduction in SSP2-2.6 offsets 14% and 23% of the negative impacts of mitigation, respectively (Supplementary Fig. 12). In sub-Saharan Africa, ozone reduction benefits for major crops are less pronounced than those in India, resulting in a smaller decrease in hunger risk in both scenarios. Climate and ozone concentration change also impact livestock prices and consumption, in turn affecting hunger risk (Supplementary Fig. 13).

### Impact of socio-economic conditions and ozone pollution levels

**Fig. 5:**
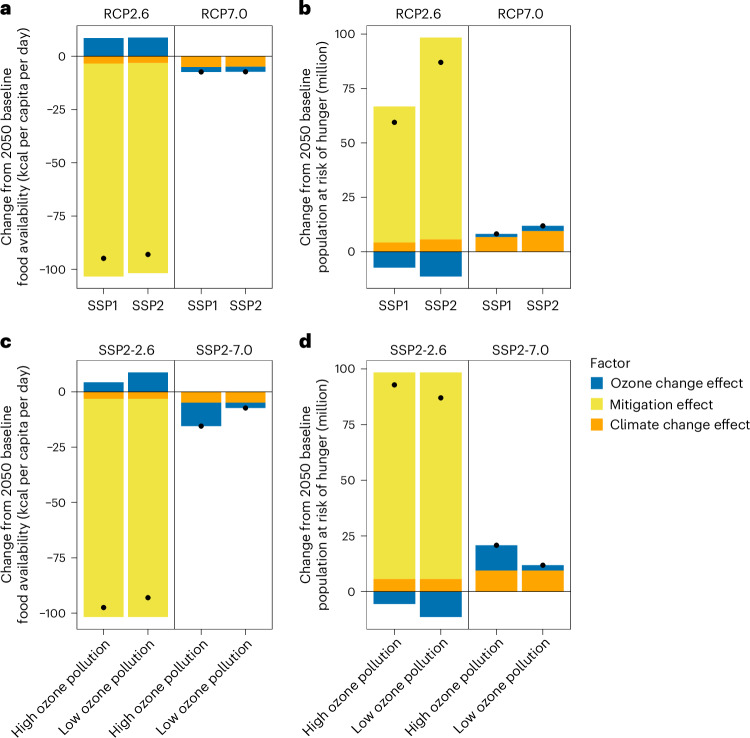
Socio-economic and air quality uncertainties in food security. **a**,**b**, Projected changes in food availability (**a**) and the population at risk of hunger (**b**) from the baseline scenario under SSP1 and SSP2 socio-economic assumptions in 2050, resulting from each of the three factors. **c**,**d**, Changes in food availability (**c**) and the population at risk of hunger (**d**) under higher ozone pollution levels than those (low ozone pollution in SSP2-2.6 and SSP2-7.0) used in our earlier results. These high ozone pollution levels also result from climate change mitigation measures; however, air pollutant co-emissions are higher than those used in our main results, yet still lower than those in the baseline scenario. The black dots indicate the combined effects of these three factors.

Socio-economic conditions and air pollution control levels could change the experimental conditions, which might change the main results. To explore this, we conducted two sets of sensitivity analyses using the AIM-Hub model. First, we assessed the consequences of using an alternative SSP (SSP1)^49^, projecting lower population growth and higher economic growth than our default experiments using SSP2. Food availability is expected to increase due to higher income growth and consequently lower the hunger risk together with lower baseline populations in both RCP scenarios. Consequently, mitigation efforts under SSP1 in 2050 lead to a smaller increase in hunger risk (67 million) than those under SSP2 (93 million) (Fig. 5 and Supplementary Fig. 14). The hunger risk decrease due to ozone reduction is also smaller, alleviating hunger by 7.1 million people compared with 11 million in SSP2, thereby offsetting 12% of the negative mitigation impacts.

Second, we included a scenario with higher ozone pollution (Supplementary Fig. 15) than that used in our main analysis. In SSP2-7.0, higher ozone pollution exacerbates the decline in food availability, resulting in an increased risk of hunger. In contrast, under SSP2-2.6, the reduction in ozone concentrations is smaller, contributing less to the alleviation of the negative impacts of mitigation. These two sensitivity analyses further support our earlier findings that ozone reduction could offset a substantial portion of the negative impacts of mitigation; however, by 2050, the resulting hunger risk will remain higher than that from warming.

## Discussion

We reevaluated the impacts of climate change and mitigation efforts on global food security by incorporating the impacts of ozone concentration changes on crop yields. Our study has two main implications that advance the current literature on mitigation and food security. First, ozone reduction is expected to reduce hunger worldwide. Future assessments of climate mitigation should account for this accompanying ozone reduction effect to better assess the benefits of climate action. Second, even after accounting for ozone reduction, climate mitigation measures may still adversely affect food security.

While we confirmed the validity of the sensitivity analysis conclusion, our study has several uncertainties regarding our results. First, we used SSP2-7.0 as a business-as-usual scenario to reflect current trends. However, other climate forcing pathways, such as the lower-emission SSP2-4.5, may better represent the recent developments^50^. Using SSP2-4.5 would probably reduce differences in climate impacts and ozone concentrations between the business-as-usual and mitigation scenarios. Nonetheless, until 2050, the emission differences between RCP4.5 and RCP7.0 remain relatively modest^50^, and the differences in land use and bioenergy deployment may also be limited^51^. The results for RCP4.5 and RCP7.0 may therefore become similar, and our main conclusion that stringent mitigation efforts may threaten food security would probably remain unchanged.

Second, we used a single ozone exposure metric, AOT40 (refs. ^21,48^), to quantify ozone-induced crop yield changes. AOT40 is a widely used metric, but other available metrics, such as M7 and M12, may produce different impact levels on crop yield^20,21^. Compared with other metrics, AOT40 generally results in a larger yield loss for wheat and rice globally in response to ozone pollution, while indicating moderate losses for maize and soybean^20^. This suggests that the offsetting effects of ozone reduction on food security may be smaller when alternative metrics are used. Even so, our qualitative conclusions about the spatial pattern and overall direction of ozone impacts remain unchanged. Separately, we assumed additive effects of ozone concentration changes and warming on crop yield, although crop sensitivity to ozone pollution can vary nonlinearly with temperature^52^. We also did not explicitly model meteorological drivers such as precipitation, humidity and solar radiation that can influence ozone concentrations^53^. Previous studies have shown that climate-driven changes in ozone are relatively minor compared with emission-driven changes^10^. These omissions are therefore more likely to adjust magnitudes than overturn our main conclusions.

Third, an additional source of uncertainty relates to our treatment of the Earth system model framework. Our simulations relied on a single atmospheric chemical transport model, which does not capture the broader spread of structural and parametric uncertainty that would be reflected in a multi-model ensemble. Discrepancies among chemical transport models can arise from differences in diagnosed model variables, such as vertical turbulent mixing, boundary layer height and vertical resolution^54^, as well as from internal physical and chemical process representations that lead to divergent outputs even under identical inputs^55^. Model uncertainty may also stem from differences in how various chemical mechanisms classify ozone chemical regimes^56^. Future studies should employ multi-model approaches to achieve more robust and comprehensive assessments. In addition, our modelling framework operates in an offline manner, in which the climate, atmospheric chemistry and crop components are not dynamically coupled. Future developments towards closer coupling among these components may enable a more integrated representation of secondary impact pathways within Earth system model frameworks.

Fourth, the contribution of each factor to hunger risk varies notably across global agricultural economic models^57^. For some models, such as ENVISAGE, climate mitigation policies primarily focus on CO_2_ emissions from fossil fuels, limiting their food availability impacts since agriculture and land use are not directly targeted. Models with broader mitigation coverage, such as AIM-Hub and GCAM, explicitly account for agricultural and land-use emissions, creating stronger linkages between mitigation policies and food availability. Carbon price differences also influence food security and may stem from differences in the representation of mitigation technologies, carbon budget assumptions or time horizon considerations. Moreover, partial equilibrium models, such as GLOBIOM and IMPACT, assume fixed incomes, emphasizing the direct effects of higher carbon prices on food systems. Computable general equilibrium models, such as AIM-Hub and MAGNET, incorporate broader economic feedback, such as income shifts and structural changes, which also influence hunger risk. Model uncertainties may also stem from regional heterogeneity, the weighting of regions in aggregation, and variations in trade volumes and calorie differences between traded and domestically consumed foods. However, our main conclusion remains unchanged regardless of the model, and the general consistency of these results should be viewed as a testament to the robustness of different climate policy impacts on hunger risk.

Our analysis did not consider alternative mitigation pathways, such as rapid renewable energy deployment or geoengineering, which could have different consequences for food security. In addition, we did not account for the health benefits of improved air quality, which can reduce disease burdens, enhance labour capacity and lower household costs^58^, thereby improving food affordability. Future assessments should also incorporate these benefits to provide a more comprehensive picture of the food security implications of climate policy.

A central policy-relevant implication of our study is that food security outcomes under climate mitigation cannot be understood by considering climate change, land-use change or ozone damage to crops in isolation. Climate change increases crop yield losses, and land-use shifts towards afforestation and bioenergy reduce cropland availability, while ozone reduction accompanying mitigation alleviates part of these losses. Integrating these three drivers provides a clearer and more policy-relevant picture of the trade-offs and offsetting effects of mitigation. For policymakers, this means the design of mitigation strategies must extend beyond carbon accounting to explicitly include their food security consequences. Strategies such as enhancing agricultural productivity^59^, achieving more efficient land-use allocation^60^, replacing red meat with other protein sources^61,62^ and improving food distribution while reducing food waste^63^ could help offset the negative impacts of climate change mitigation on food security and support progress towards the zero-hunger goal.

## Methods

Here we used six global agro-economic models to examine how climate change, emission mitigation efforts and the accompanying ozone reduction from climate mitigation interact to affect global food security in various climate policy scenarios. All models represented agricultural markets, land use and emissions at varying levels of detail. The overall research framework is shown in Fig. 1. We first used the AIM modelling framework and an atmospheric chemical transport model to compute ozone concentration changes under different climate conditions, and then calculated ozone-induced crop yield losses on the basis of ozone-exposure–yield-loss functions. We then input the estimated crop yield changes, along with potential climate-induced crop production from crop model outputs, and future land-use information into each agro-economic model. For each climate policy scenario, each model directly outputs the calorie intake by region, which was then used to estimate the population at risk of hunger.

### Modelling experiment

We developed six scenarios combining climate change, stringent mitigation policies aligned with the 1.5 °C Paris Agreement goal^5^ and the accompanying ozone reduction from climate change mitigation efforts to examine their effects on global food security (Table 1). Our analysis is based on the SSP–RCP framework developed for the Coupled Model Intercomparison Project Phase 6, which integrates SSPs with RCPs^43^. To capture a range of plausible futures, we used SSP2, a “middle-of-the-road” socio-economic development pathway^44^, combined with two contrasting radiative forcing trajectories: RCP2.6 and RCP7.0. Under SSP2, population growth is moderate, and social, economic and technological trends follow historical patterns in each region^44^. The variables under the SSP2 storyline such as gross domestic product and population, obtained from the SSP database^64^, were used in a common simulation protocol to force individual models. Other characteristics of SSP2, such as technological development, dietary patterns, land-use change and international trade, were implemented by each model according to its own assumptions. RCP2.6 represents ambitious mitigation efforts aimed at limiting warming to below 1.5 °C by 2100. In contrast, RCP7.0, a high-emissions scenario, reflects minimal mitigation based on NPis, such as renewable energy targets and coal phase-out plans. The policy cut-off year is 2020, although the continuation year varies across the models. These combinations—SSP2-2.6 and SSP2-7.0—represent mitigation and business-as-usual futures, respectively.

For comparison, we also developed a baseline scenario (BAU_NoCC in Table 1) that assumes that the current climate change and ozone air pollution conditions prevail. All non-mitigation scenarios (BAU_NoCC, BAU and OZBAU in Table 1) were aligned with minimal mitigation efforts, consistent with the NPis. The decomposition method used to separate the climate change effects, mitigation and ozone reduction is presented in Supplementary Table 1. The difference between climate change scenarios (BAU, BAU_MITI and BAU_MITI_RCP26) and the baseline scenario (BAU_NoCC) shows the pure effects of climate change. The difference between scenarios with (BAU_MITI) and without (BAU) stringent climate change mitigation enables the assessment of the mitigation effects. Comparing the scenarios with (OZBAU_MITI_RCP26 and OZBAU) and without (BAU_MITI_RCP26 and BAU) ozone reductions under the same conditions allows for assessments of ozone concentration changes.

### Model descriptions

The global agro-economic models used in our analysis integrate economic and land-use components to capture interactions across sectors and regions. They represent agricultural and land systems in detail, simulating how climate policies affect crop yields, trade patterns and GHG emissions, including ozone precursors. By incorporating climate mitigation scenarios, such as bioenergy expansion and land-use changes, along with the accompanying ozone reduction, the models enable a comprehensive assessment of potential trade-offs and synergies between climate goals and food security.

The AIM-Hub model, formerly known as AIM/CGE^35^ (Asia-Pacific Integrated Model/Computable General Equilibrium), is a one-year-step recursive-dynamic general equilibrium model that has global coverage, including 17 regions and 42 industrial classifications, with detailed agricultural sectors to assess bioenergy and land-use competition accurately. The model maximizes production sector profits using multi-nested constant-elasticity-of-substitution functions for input prices and models household expenditures with a linear expenditure system function, updated recursively by income elasticity. It includes GHGs such as CO_2_ from energy and non-energy sources, CH_4_, N_2_O and fluorinated gases. Energy-related emissions are linked to fossil fuel use, while non-energy CO_2_ emissions stem from land-use changes and industrial processes, with land-use-change emissions calculated on the basis of forest area changes and carbon stock density differentiated by global agro-ecological zones. Non-energy emissions from other sources are proportional to each related activity level. CH_4_ emissions arise from rice production, livestock, fossil fuel mining and waste management, and N_2_O emissions arise from fertilizer application, livestock manure management and the chemical industry.

The ENVISAGE (Environmental Impact and Sustainability Applied General Equilibrium) model^36^ is a global computable general equilibrium model that operates within a recursive dynamic framework, modelling the circular flow of an economy. The global economy in the model is represented by 14 regions and 36 activities. Firms produce goods and services by purchasing input factors such as labour and capital, while households receive income from these factors and, in turn, demand goods and services. The model balances supply and demand to determine equilibrium prices. Production is characterized by constant-elasticity-of-substitution functions, capturing input substitutability across sectors, including crops, livestock and general capital/labour dynamics. ENVISAGE integrates various household demand functions, such as the linear expenditure system and constant differences in elasticity. It uses an Armington specification for trade, differentiating goods by region of origin, and includes detailed trade and price mechanisms. The model features segmented labour markets, partially mobile capital and logistic supply curves for land and water. It also incorporates GHG emissions, allowing for carbon taxes, emission caps and permit trade systems. Dynamics are driven by labour supply growth, capital accumulation and technological change, with a possibility to link labour productivity to research and development expenditure.

GCAM (the Global Change Assessment Model)^37,65^ is an integrated assessment model that links representations of the global economy, energy systems, agriculture and land use, water and climate. GCAM divides the world into 32 geopolitical regions and represents land at the subregional level across 235 water basins. The agriculture and land-use modules simulate the supply, demand and trade of crops, livestock, forestry products and bioenergy, with land allocated among competing uses on the basis of profitability, including natural and non-commercial land categories. GCAM tracks GHG emissions across sectors and provides internally consistent projections of emissions, land use and energy system transitions. Carbon prices, when applied, influence energy technology costs and the deployment of land-based mitigation measures, thereby shaping land-use decisions. This study employs GCAM version 7.1, with essential modifications to represent agricultural labour markets and food waste pathways, as well as to incorporate harmonized agricultural productivity drivers from AgMIP.

The GLOBIOM^38^ (Global Biosphere Management) model is a recursive dynamic partial equilibrium model that covers agricultural, forestry and bioenergy sectors, incorporating detailed biophysical constraints, technological costs and environmental parameters, including comprehensive GHG emission accounts and irrigation water use. It represents commodity markets and international trade across 59 economic regions. GLOBIOM projects demand for commodities and trade flows on the basis of population, income, production costs and prices. It uses biophysical data to account for spatial heterogeneity in productivity and allocates resources to maximize social welfare. Under a global carbon pricing framework, GLOBIOM achieves mitigation by incentivizing structural changes in agriculture and trade, implementing various mitigation options and influencing food demand adjustments to reduce emissions.

The IMPACT^39,66^ (International Model for Policy Analysis of Agricultural Commodities and Trade) model is a system of interconnected economic and biophysical models that operates within a recursive dynamic partial equilibrium multi-market framework. It simulates both national and international agricultural markets, analysing commodity production, demand and trade across 159 countries for 62 agricultural commodities, including crops, livestock products and processed goods such as refined sugar and food oils. Agricultural production is modelled within 320 food production units, which are geographical units defined by national boundary and hydrological basin overlaps. This set-up allows for integration with hydrology and water resource management modules, enabling the assessment of how changes in water availability affect irrigation.

MAGNET (the Modular Applied General Equilibrium Tool)^40^ is a multi-regional, multi-sectoral, applied general equilibrium model based on neo-classical microeconomic theory. It extends the standard Global Trade Analysis Project model and covers 161 regions and 62 agricultural sectors. It features an input–output model that connects industries through value-added chains from primary goods to final goods and services for consumption, with input and output prices determined endogenously to balance supply and demand. The agricultural sector in MAGNET is represented in high detail compared with standard computable general equilibrium models. Productivity changes are driven by a combination of autonomous technological advancements provided by the IMAGE^67^ model and economic processes modelled by MAGNET, such as factor substitution. Land is explicitly modelled as a production factor with a supply curve based on land availability data from IMAGE, a comprehensive integrated assessment framework that models interactions between humans and natural systems. It includes sub-models for land use, agricultural economy, energy systems, natural vegetation, hydrology and climate systems.

### Implementation of climate change mitigation

Ambitious mitigation measures and cost-effective emission reductions were achieved by applying a uniform global carbon price on GHG emissions across agriculture, land use and other sectors in most of the agro-economic models. The carbon price was set to that for mitigation accompanied by an EAT-Lancet-style food system transformation^41,42^, with mitigation policies aligned with the 1.5 °C target.

The implementation details vary depending on the structure and capabilities of each model. AIM implements emission mitigation by setting a carbon budget of 500 GtCO_2_ for this century. For non-CO_2_ emissions, a marginal abatement cost curve is applied^68^. In ENVISAGE, mitigation is based on CO_2_ carbon budgets estimated by Chepeliev et al.^69^. However, the carbon price is applied only to CO_2_ emissions from fossil fuel combustion, whereas changes in non-CO_2_ emissions result from dietary shifts and reductions in food loss and waste. A carbon price path in the GCAM model was used, starting in 2025, fully phased in by 2035 and increasing at a 3% Hotelling rate, under which the end-of-century temperature stabilizes around 1.5 °C. In GLOBIOM, carbon prices are applied to both CO_2_ and non-CO_2_ emissions across the agriculture, forestry and land-use sectors. In IMPACT, mitigation is driven by an exogenous land growth driver. In MAGNET, a carbon tax of US$100 per tonne CO_2_-equivalent emissions is applied to both CO_2_ and non-CO_2_ emissions by 2035, along with the implementation of marginal abatement cost curves in the agricultural sectors. Carbon pricing revenue is returned to households through lump-sum recycling.

This raises production and food costs through three main channels: increasing costs based on agricultural GHG intensity, making cropland expansion more expensive and boosting biomass demand, which raises land rents. The higher costs lead to increased food prices and reduced consumption. In the general equilibrium models—AIM-Hub, ENVISAGE and MAGNET—there was also an income channel playing a crucial role in food affordability and the purchasing power of consumers. This channel showed how changes in income from mitigation policies or economic shifts affect the ability of households to buy food, especially when production and food costs rise. Carbon prices may also drive renewable energy adoption, energy substitution with capital, carbon capture and storage technology, and other mitigation technologies. Ozone reduction is an accompanying effect of climate change mitigation efforts—reduced reliance on fossil fuels and increased electric vehicle use—lowering air pollutant co-emissions, including ozone precursors such as CH_4_, NO_*x*_ and VOCs (Supplementary Fig. 4).

### Projection of ozone concentration

Ozone concentrations under each RCP are projected using the GEOS-Chem (Goddard Earth Observing System-Chemistry) global three-dimensional model of atmospheric chemistry and transport (version 13-04)^70^. The meteorological data and anthropogenic air pollutant emissions under each scenario are used as inputs to drive this model. Here we utilized the full chemistry mechanism, incorporating a detailed chemical scheme to simulate ozone production and destruction, particularly focusing on O_*x*_–NO_*x*_ chemistry, as outlined by Mao et al.^71^ and Parrella et al.^72^.

For natural emissions relevant to ozone formation, we used archived inventories from the Harmonized Emissions Component. These inventories include NO_*x*_ emissions from lightning, biogenic VOC emissions and other natural sources such as dust and sea salt emissions, as well as volcanic eruptions^73,74^. The meteorological data come from the NASA Global Modelling Assimilation Office MERRA2 reanalysis meteorological data product^75^, which aggregates data at a spatial resolution of 0.5° × 0.5° across 72 vertical layers. Anthropogenic air pollutant emissions, including CO_2_, N_2_O, black carbon, carbon monoxide, ammonia, CH_4_, non-methane VOCs, NO_*x*_, organic carbon and sulfur oxides, are derived from the AIM-Hub model^35^. To account for future methane trends, we conducted simulations using scenario-specific CH_4_ concentrations, assuming a well-mixed atmosphere. Specifically, we used two datasets to derive monthly gridded CH_4_ concentration fields. One was the yearly global mean CH_4_ concentration computed by the climate emulator MAGICC with the inputs of the AIM-Hub GHG and air pollutant emissions scenarios. The second dataset was the grid-wise 2016 monthly CH_4_ data at 1° × 1° resolution from NASA. We simply applied scaling factors to the monthly gridded concentration, which was taken from the rate of change of the global mean concentration in 2016 for future years. This method preserves the spatial variability of CH_4_ while aligning concentrations with the narrative of each scenario. These derived emissions are influenced by changes in factors such as food demand and supply, technological developments, renewable energy adoption and land-use changes as part of climate change mitigation efforts. For example, coal and other fossil fuels remain notable contributors to emissions, but their role is diminished with the transition to cleaner energy sources, including renewable energy and carbon capture technologies, which contribute to achieving long-term emission reduction targets and reducing associated air pollutants.

Since the AIM-Hub model outputs a regionally aggregated emission inventory across 17 regions, we used the AIM Downscaling Tool^76^ to generate gridded emissions at a resolution of 0.5° × 0.5° for use in the GEOS-Chem model. The simulated surface ozone concentrations were then generated at a resolution of 2° × 2.5° for the period from 2005 to 2050, with 2005 also chosen for the model calibration. In our baseline scenario, climate conditions and ozone concentrations were fixed at the current levels.

### Estimation of crop yield changes due to ozone reduction and climate change

The baseline crop yield was projected by the IMPACT model, which provides crop yield estimates for 37 crops across 159 countries/regions^39^. Crop (maize, rice, wheat and soybean) yield changes from the baseline scenario under climate change scenarios SSP2-2.6 and SSP2-7.0 were obtained from the Global Gridded Crop Model Intercomparison Phase 3 of AgMIP, with detailed projection methods described by Jägermeyr et al.^45^. We used crop yield simulations from an ensemble of crop models; however, it should be noted that there is uncertainty in the climate impacts depending on the underlying global climate model used^45^. These four crops were mapped to the 37 crop commodities in the IMPACT model, following a previous mapping method^77^, to derive yield changes due to climate shocks. For the ozone-induced crop yield changes, we used the ozone exposure metric AOT40—a widely used measure for assessing the impact of surface ozone pollution on vegetation, including crops and forests—and expressed this as equation (1)^21,48^:

1 $$\begin{array}{l}\mathrm{AOT}40\,(\mathrm{ppmh})=\mathop{\sum }\limits_{i=1}^{n}\max ({[{{\rm{O}}}_{3}]}_{i}-\mathrm{thres},\,0),\\ \,\,\,\,\,\,\,\,\,\,\,\,\,\,\,\,\,\,\,\,\,\,\,\,\,\,\,\,\,\,\,\,\,\mathrm{thres}=40\,\mathrm{ppbv}\,(08:00\mbox{--}19:59)\end{array}\,\,\,$$

where AOT40 is the cumulative sum of ozone concentrations exceeding the threshold of 40 ppbv during daylight hours (08:00–19:59) over a three-month growing season at each grid level, [O_3_]_*i*_ is the hourly average ozone concentration and *n* is the number of hours counted during the crop growing season. The obtained AOT40 values were then used to calculate crop-specific relative yield loss (RYL) due to ozone exposure using exposure–response functions (ERFs) from Van Dingenen et al.^21^ and Mills et al.^48^. The ERFs directly express RYL as a function of AOT40 for each crop. Because the original AOT40-based ERFs in Mills et al.^48^ imply a non-zero RYL at AOT40 = 0, we rescaled those ERFs to a zero-intercept form following Van Dingenen et al.^21^, so that RYL = 0 at zero ozone exposure. The crop-specific coefficients after rescaling (and the ERF function used for each crop) are listed in Supplementary Table 2.

We overlayed the climate- and ozone-induced crop-specific yields with future land-use grid data^76^, which were then aggregated into 17 global regions to derive regional yield changes (Supplementary Tables 3 and 4), which were input into the global agro-economic models to generate final outputs for food-related indicators, such as crop production, agricultural commodity prices and calorie intake. Mitigation does not affect these exogenous crop yields in any of the agro-economic models except for IMPACT, which is also linked to a water model. Water shocks, resulting from differences in land growth rates, could affect crop yields (Supplementary Fig. 5).

### Estimation of populations at risk of hunger

The population at risk of hunger is estimated on the basis of the Food and Agriculture Organization’s (FAO) methodology^78^, which has been applied in previous studies^32–34^. Hunger is defined as a state of energy (calorie) deprivation lasting over one year. This definition excludes the short-lived effects of temporary crises and does not account for the inadequate intake of other essential nutrients^79^. It is calculated as follows (equations (2)–(7)):

2 $${{\rm{Risk}}}_{t}={{\rm{POP}}}_{t}\times {{\rm{PoU}}}_{t}$$

where Risk_*t*_ is the population at risk of hunger in year *t*, POP_*t*_ is the population in year *t* and PoU_*t*_ is the proportion of the population at risk of hunger in year *t*. The population data were obtained from the SSP database^64^. PoU_*t*_ was estimated using three parameters: the mean food calorie consumption per capita per day (cal_*t*_), the mean minimum dietary energy requirement (*M*_*t*_) and the coefficient of variation (CV) of the distribution of dietary energy consumption in a country. The food distribution within a country was assumed to follow a lognormal distribution, determined by the mean food calorie consumption per capita per day and the equity of food distribution (CV). The proportion of the population below *M*_*t*_ was then defined as PoU. The mean food calorie consumption per capita per day was output from each model. We aligned each model’s mean food calorie consumption per capita per day in the base year with the values reported by the FAO. We then applied the rate of change from the base-year values relative to the FAO values to all subsequent years. The adjusted mean food calorie consumption for each model was then used in the calculation of the risk of hunger. The CV is an indicator of food security determined by the FAO through household surveys and ranges from 0 to 1. We weighted the CV data for each FAO country on the basis of base-year population data and aggregated them by region to produce a regional CV. The CV changes over time and decreases with future income growth^32^. Specifically, PoU_*t*_ is calculated as follows:

3 $${\mathrm{PoU}}_{t}=\Phi \left(\frac{\log \,{M}_{t}-\mu ({\mathrm{cal}}_{t},{\sigma }_{t})}{{\sigma }_{t}}\right)$$

4 $$\mu ({{\rm{cal}}}_{t},{\sigma }_{t})=\,\log \,{{\rm{cal}}}_{t}-{\sigma }_{t}^{2}/2$$

5 $${\sigma }_{t}={[\log ({{\rm{CV}}}^{2}+1)]}^{0.5}$$

where Φ is the standard normal cumulative distribution. For *M*_*t*_ in the base year, we used the value estimated by the FAO. We adjusted the M_*t*_ in each year after the base year using the adjustment coefficient for the minimum dietary energy requirement per capita in different age and sex groups^80^ and the population size of each age and sex group in each year^64^, as expressed in the following equations:

6 $${M}_{t}={M}_{\mathrm{base}}\times \frac{{\mathrm{MER}}_{t}}{{\mathrm{MER}}_{\mathrm{base}}}$$

7 $${\mathrm{MER}}_{t}=\frac{{\sum }_{i,\,j}\left({\mathrm{RMER}}_{i,\,j}\times {P}_{{\mathrm{class}}_{i,\,j,t}}\right)}{{\sum }_{i,\,j}{P}_{{\mathrm{class}}_{i,\,j,t}}}$$

where *M*_base_ is the minimum dietary energy requirement per capita in the base year, RMER_*i,j*_ is the　adjustment coefficient for the minimum dietary energy requirements per capita of age *i* and sex *j*, and ${P}_{{{\rm{class}}}_{i,j,t}}$ is the population of age *i* and sex *j* in year *t*. For the hunger analysis, we divided the world into six regions—sub-Saharan Africa, China, India, Southeast Asia, other parts of Asia and the rest of the world—on the basis of current hunger risk levels^32^, surface ozone concentrations and projected ozone changes under mitigation^30^. For instance, sub-Saharan Africa and India have the largest populations at risk of hunger^32^. Regions such as China, India and sub-Saharan Africa currently experience higher ozone pollution levels, with ozone concentration changes projected to remain higher in these regions under mitigation^30^.

### Statistics and reproducibility

The study uses a scenario-based sampling strategy. Six policy scenarios (baseline and mitigation pathways consistent with SSPs and RCPs) were applied across all six models. No data were excluded from the analyses. The experiments were not randomized. The investigators were not blinded to allocation during experiments and outcome assessment. All figures were generated using R (version 4.5.0)^81^, with key packages including dplyr (version 1.1.4)^82^, ggplot2 (version 3.5.2)^83^ and tidyverse (version 2.0.0)^84^.

### Reporting summary

Further information on research design is available in the Nature Portfolio Reporting Summary linked to this article.

## Supplementary information

Supplementary Information Supplementary Figs. 1–15 and Tables 1–4.

Reporting Summary

## Data Availability

The data used to generate the figures are available via figshare at 10.6084/m9.figshare.28051235 (ref. ^85^).

## Code availability

All code needed to replicate the findings and display items of this study is available via figshare at 10.6084/m9.figshare.28051235 (ref. ^85^).
