# Supplementary material for: Ozone pollution reduction partially offsets the negative impact of climate change mitigation efforts on global hunger
Source: Nat Food. 2026 Mar 16;7(4):356–68. doi: 10.1038/s43016-026-01322-3 (PMC13121001; doi:10.1038/s43016-026-01322-3)
Supplement: Supplementary file 1 — Supplementary Figs. 1–15 and Tables 1–4. [file 43016_2026_1322_MOESM1_ESM.pdf]

nature food

Supplementary information https://doi.org/10.1038/s43016-026-01322-3

Ozone pollution reduction partially offsets the negative impact of climate change mitigation efforts on global hunger

In the format provided by the authors and unedited

Table of contents

Supplementary Fig. 1. GDP (2005 USD market exchange rate) per capita[1] in each region by 2050 in the SSP2 socioeconomic pathway[2]. 2

Supplementary Fig. 2. Projected population[1] in each region by 2050 in the SSP2 socioeconomic pathway[2]........ 2

Supplementary Fig. 3. Change in the population at risk of hunger compared to the baseline scenario under the SSP2 socioeconomic pathway[2]. 3

Supplementary Fig. 4. Changes in CH4, NOx and VOC emissions under the SSP2-2.6 and SSP2-7.0 scenarios over time, based on projections from the AIM/Hub model 4

Supplementary Fig. 5. Changes in exogenous crop yields in 2050 for each region relative to the baseline scenario 5

Supplementary Fig. 6. Projected changes in global mean food security across climate policy scenarios. ... 6

Supplementary Fig. 7. Projected changes in food security across climate policy scenarios and regions. 7

Supplementary Fig. 8. Changes in food calorie availability from the baseline scenario across climate policy scenarios........ 8

Supplementary Fig. 9. Projected changes in hunger risk across climate policy scenarios and regions. 9

Supplementary Fig. 10. Changes in crop production and crop-based food availability due to climate change and mitigation. 10

Supplementary Fig. 11. Share of the current global production volume (in tons) of major crops projected for 2050. 11

Supplementary Fig. 12. Percentage of mitigation-induced hunger risk offset by the accompanying ozone reduction in 2050. 12

Supplementary Fig. 13. Changes in global mean agricultural commodity prices (a) and calorie availability (b) of each food commodity in 2050 from the baseline scenario. 13

Supplementary Fig. 14. Socioeconomic and air quality uncertainties in food security. 14

Supplementary Fig. 15. Changes in CH4, NOx and VOC emissions under the SSP2-2.6 and SSP2-7.0 scenarios over time, based on projections from the AIM/Hub model. 15

Supplementary Table 1. Decomposition method. 16

Supplementary Table 2. Exposure-response functions (ERFs) used to evaluate the relative yield loss. 17

Supplementary Table 3. Regional categories used in the Agricultural Model Intercomparison and Improvement Project (AgMIP) 18

Supplementary Table 4. Regional mapping. 19

1. Supplementary figures

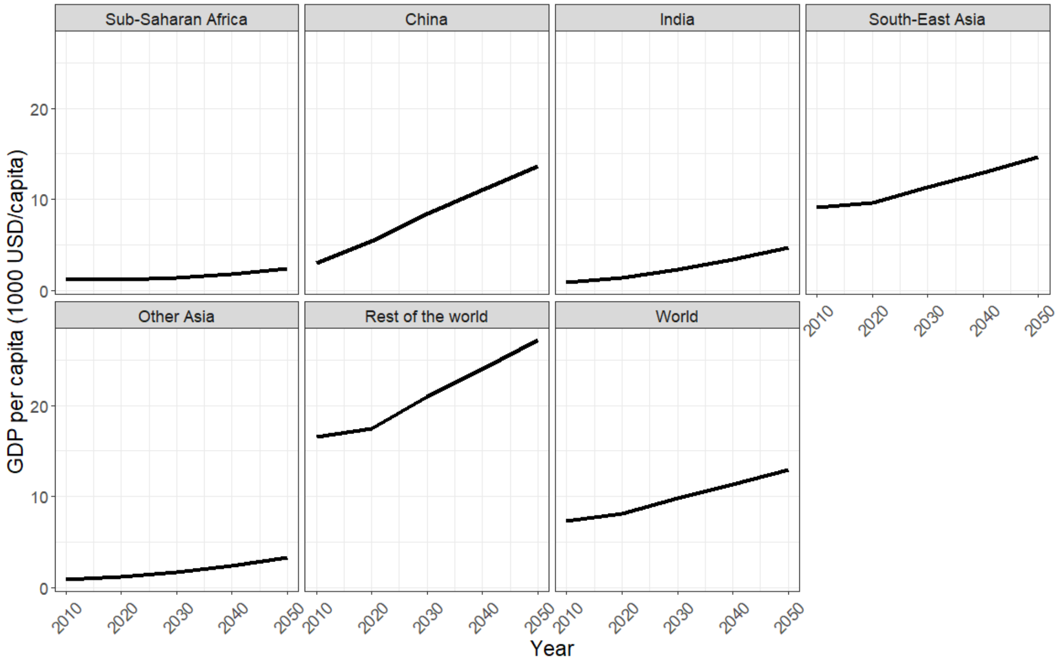

Supplementary Fig. 1. GDP (2005 USD market exchange rate) per capita[1] in each region by 2050 in the SSP2 socioeconomic pathway[2].

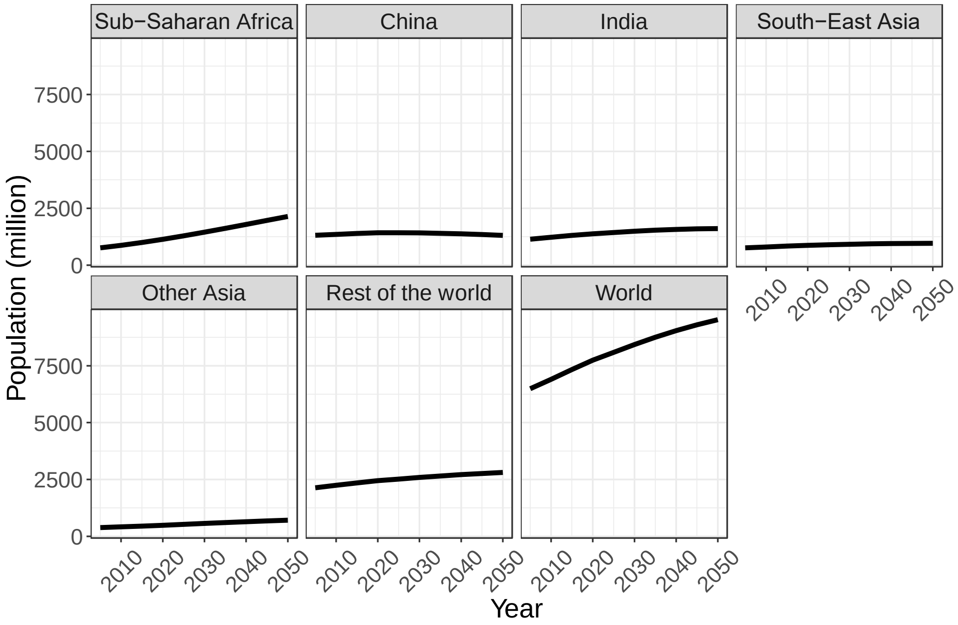

Supplementary Fig. 2. Projected population[1] in each region by 2050 in the SSP2 socioeconomic pathway[2].

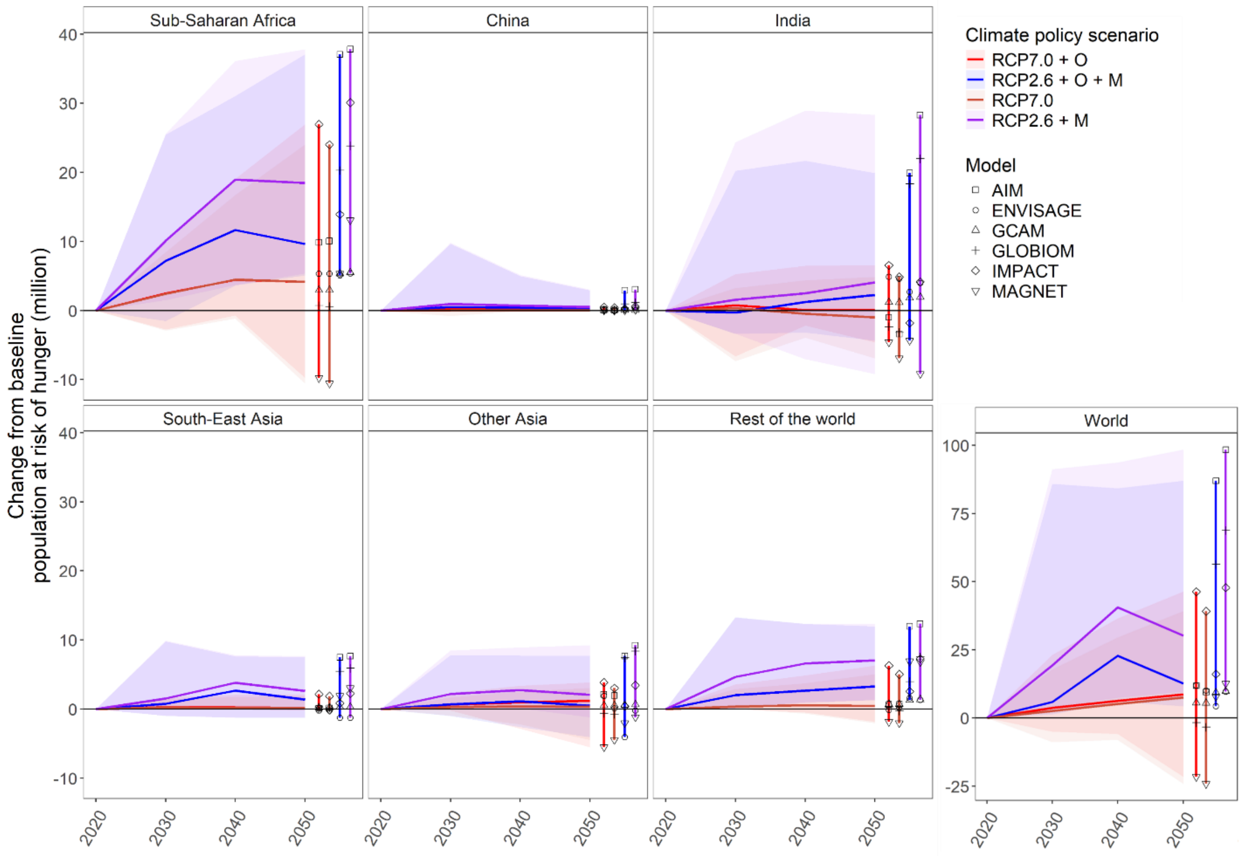

Supplementary Fig. 3. Change in the population at risk of hunger compared to the baseline scenario under the SSP2 socioeconomic pathway[2]. RCP2.6 and RCP7.0 represent the climate change effect, O represents the effect of the accompanying ozone concentration changes from mitigation or changes in air pollutant emissions, and M refers to the impact of mitigation policies, excluding the accompanying ozone reduction effect. Solid lines represent the median values across multiple models. Shaded areas indicate the ranges across the model estimates, and markers within the vertical bars indicate results from each global agricultural economic model in 2050.

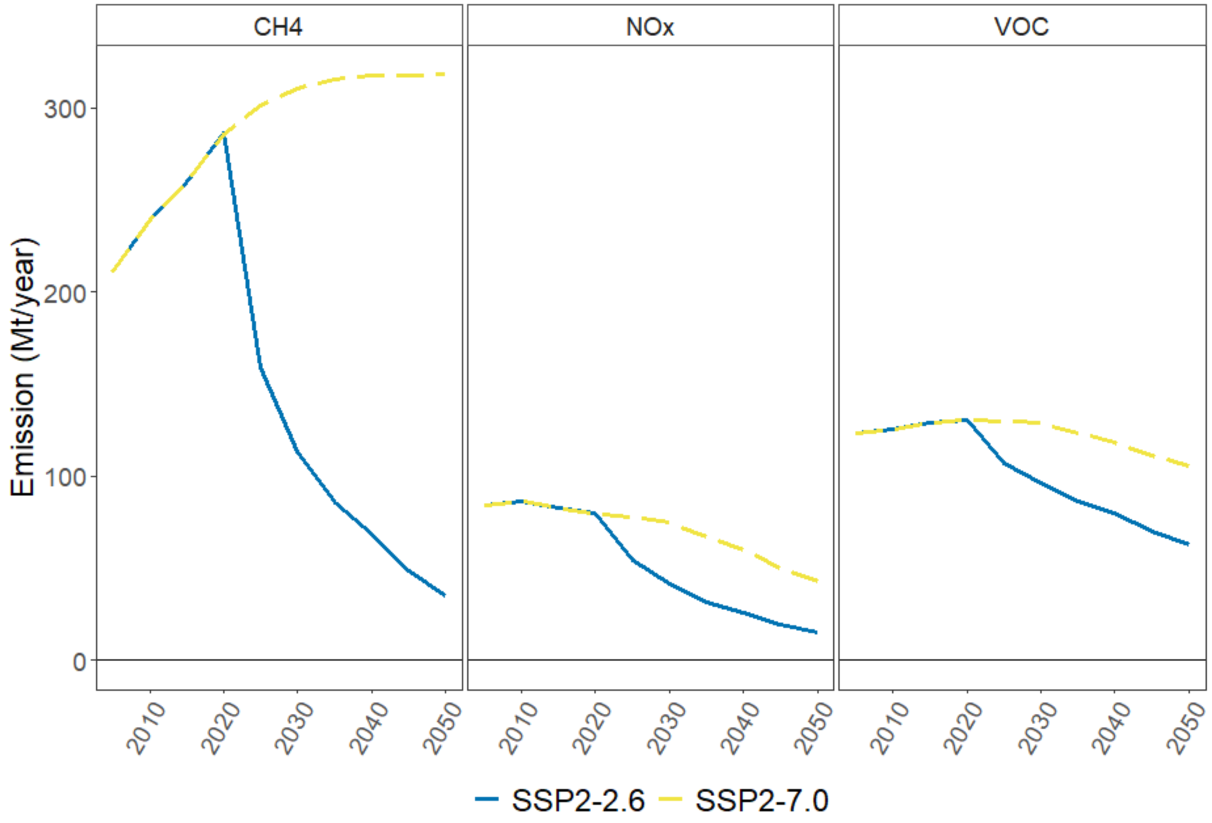

Supplementary Fig. 4. Changes in CH4, NOx and VOC emissions under the SSP2-2.6 and SSP2-7.0 scenarios over time, based on projections from the AIM/Hub model.

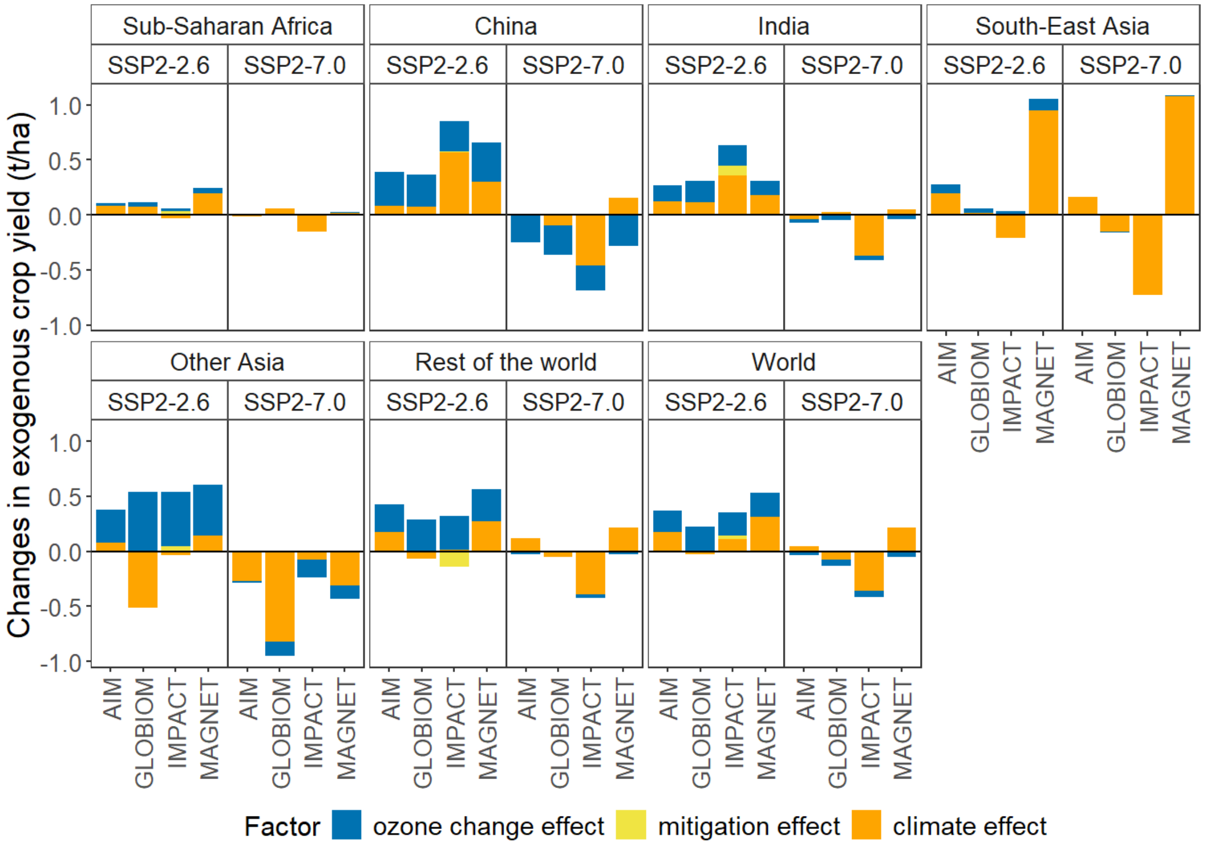

Supplementary Fig. 5. Changes in exogenous crop yields in 2050 for each region relative to the baseline scenario. The changes are driven by the accompanying ozone concentration changes resulting from air pollutant emission changes in each scenario, the negative impacts of mitigation efforts, and climate change**.** ENVISAGE and GCAM are excluded due to the lack of available data on absolute changes in crop yield.

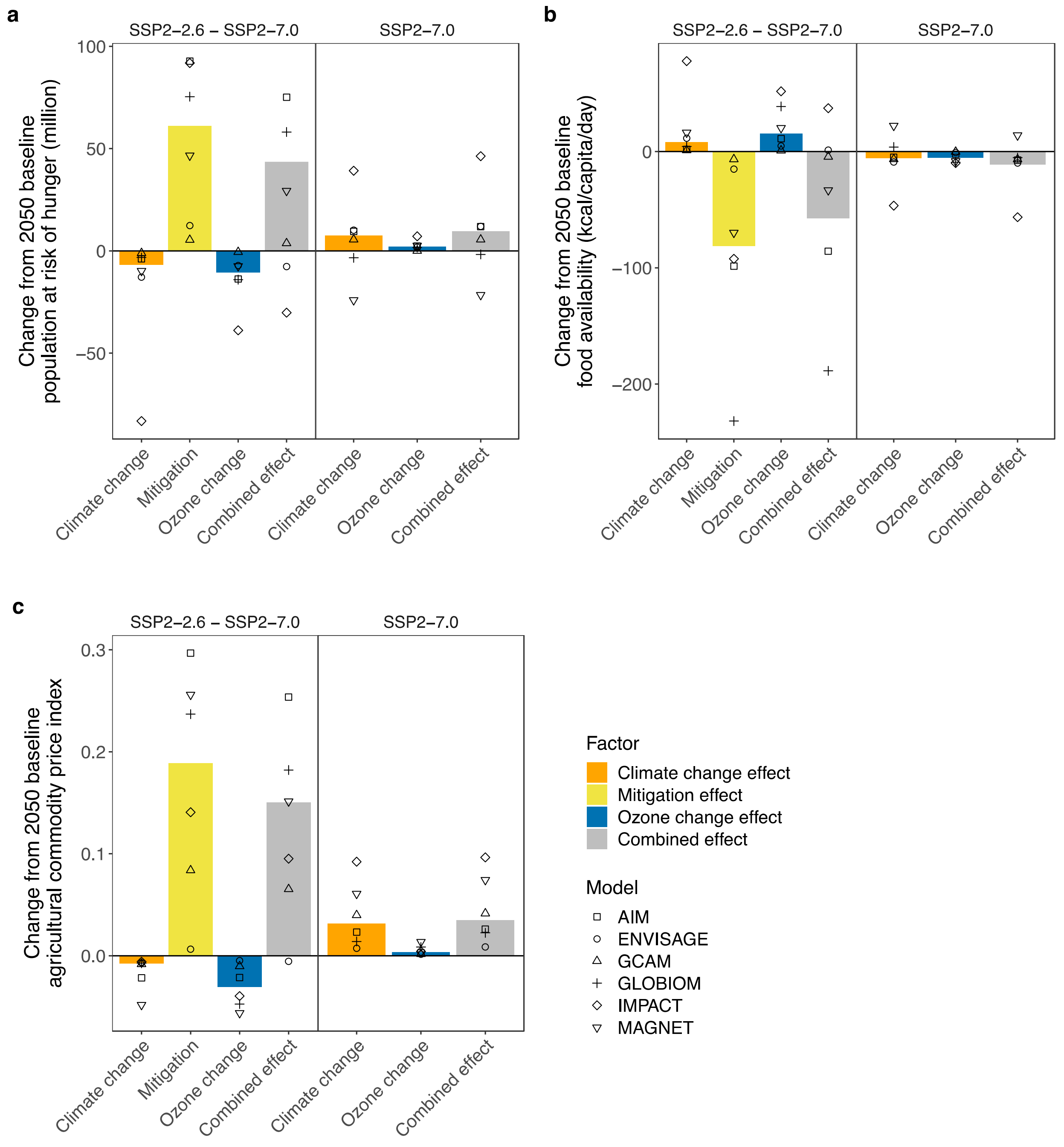

Supplementary Fig. 6. Projected changes in global mean food security across climate policy scenarios. Changes in hunger risk (**a**), food availability (**b**), and agricultural commodity price (**c**) from the baseline scenario in 2050 due to climate change, mitigation policy (excluding ozone reduction effects), and ozone concentration change, respectively. The expression SSP2-2.6 – SSP2-7.0 denotes the difference between the SSP2-2.6 and SSP2-7.0 scenarios. Bars indicate the multi-model median, whereas symbols represent results from individual global agricultural economic models.

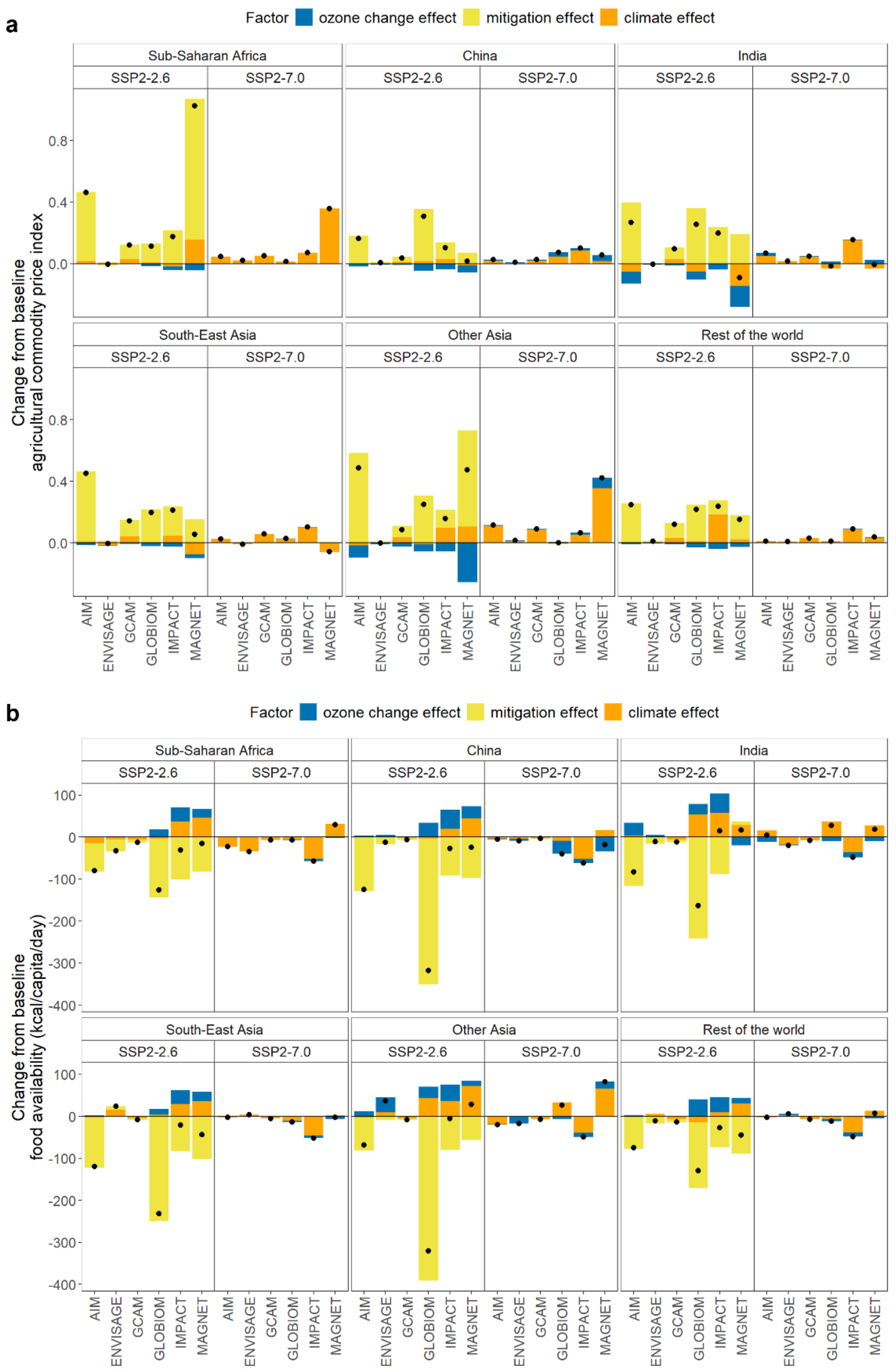

Supplementary Fig. 7. Projected changes in food security across climate policy scenarios and regions. Changes in agricultural commodity prices (**a**), and food availability (**b**) from the baseline scenario in 2050 due to climate change, mitigation policy (excluding the accompanying ozone reduction effect), and ozone concentration change, respectively. Black dots indicate the combined effects of these three factors.

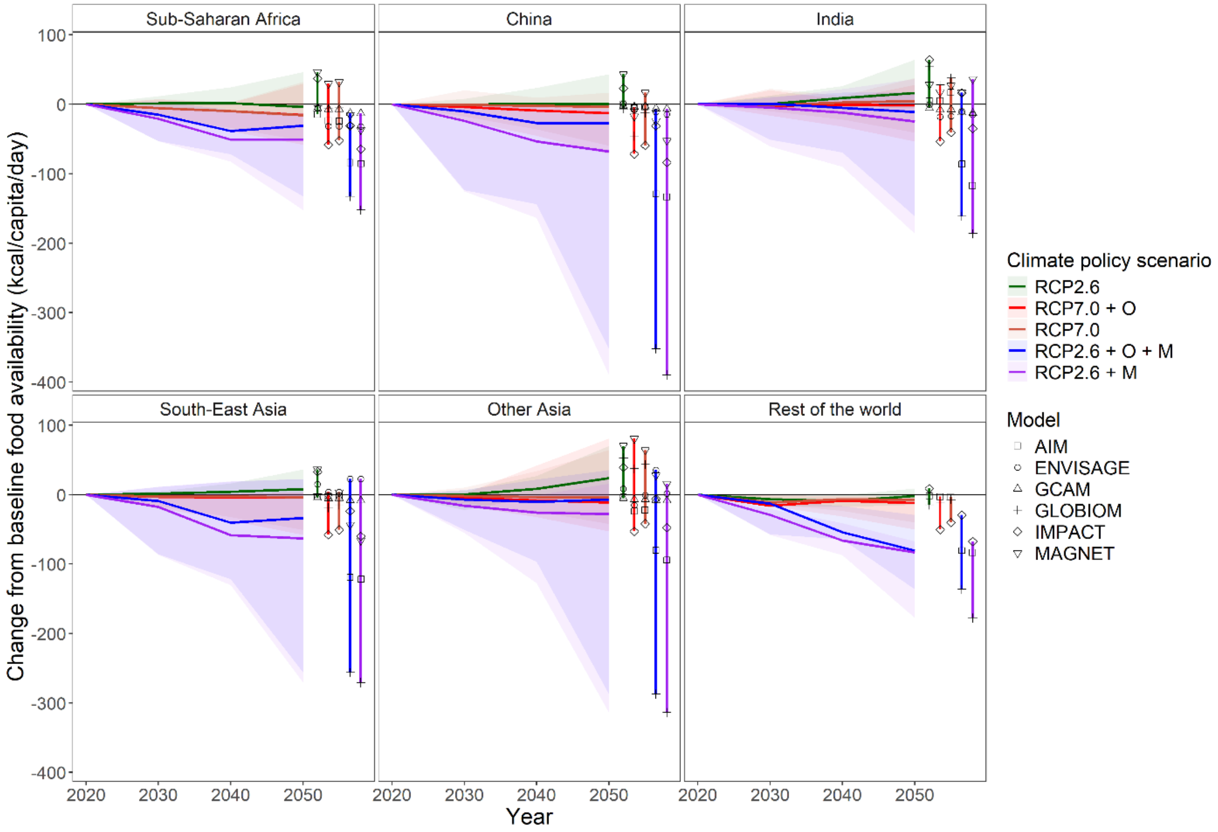

Supplementary Fig. 8. Changes in food calorie availability from the baseline scenario across climate policy scenarios. Solid lines represent the median values across multiple models. Shaded areas indicate the ranges across the model estimates, and markers within the vertical bars indicate results from each global agricultural economic model in 2050.

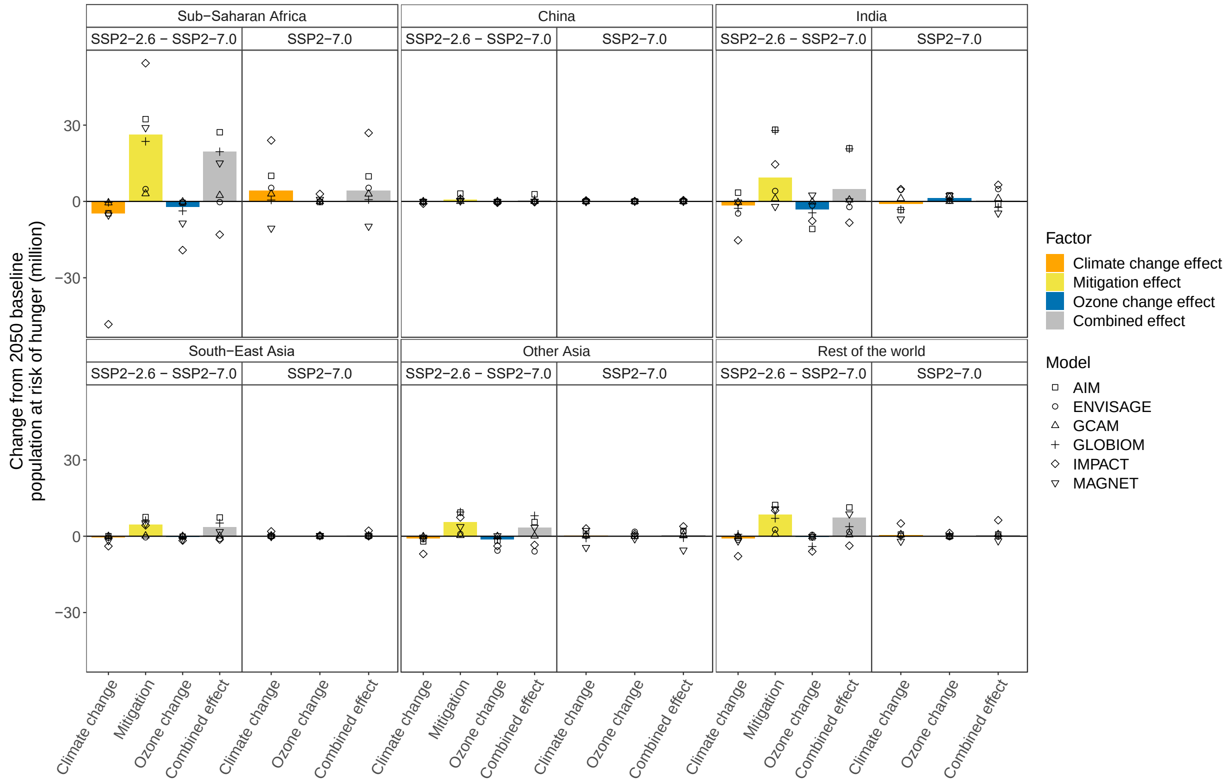

Supplementary Fig. 9. Projected changes in hunger risk across climate policy scenarios and regions. Changes in hunger risk from the 2050 baseline scenario are shown for three factors: climate change, mitigation policy (excluding the accompanying ozone reduction effect), and ozone concentration change. The expression SSP2-2.6 – SSP2-7.0 denotes the difference in outcomes between the SSP2-2.6 and SSP2-7.0 scenarios. Bars indicate the multi-model median, whereas symbols represent results from individual global agricultural economic models.

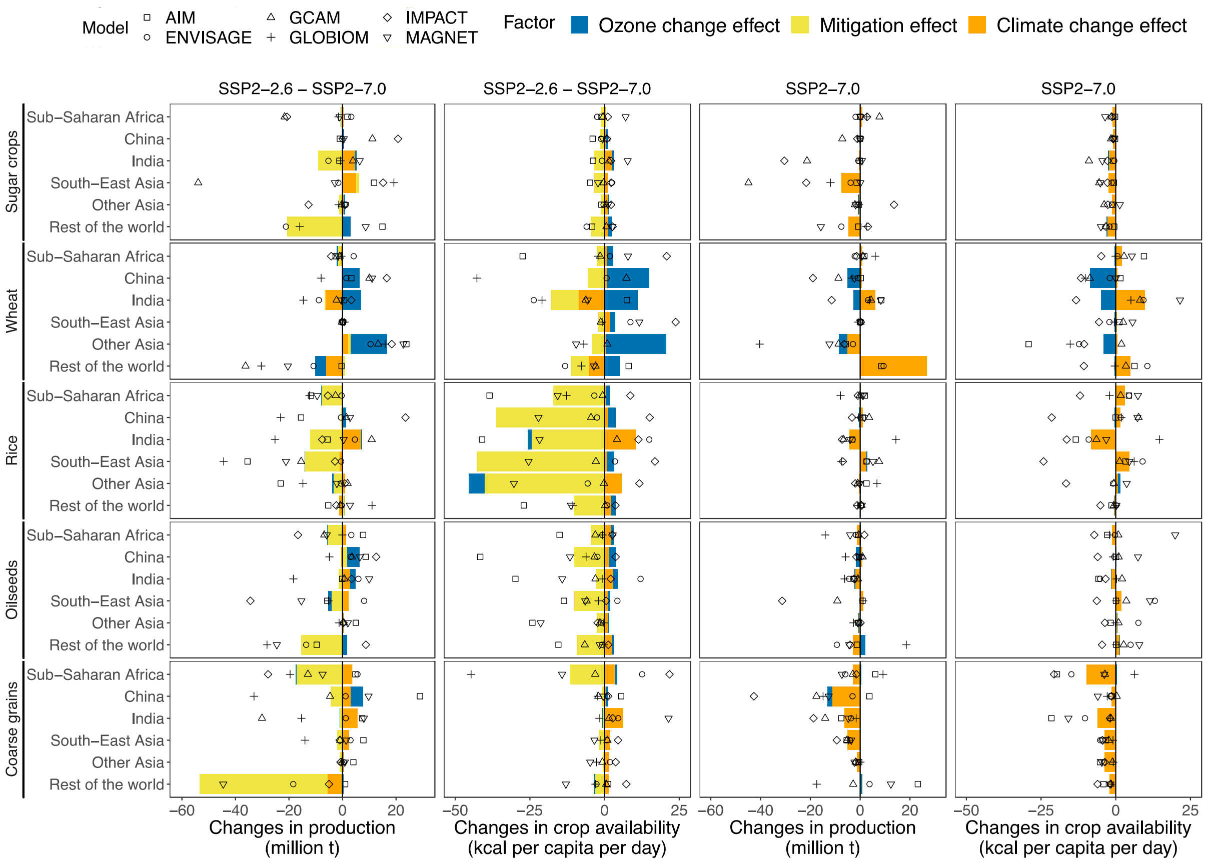

Supplementary Fig. 10. Changes in crop production and crop-based food availability due to climate change and mitigation. The expression SSP2-2.6 – SSP2-7.0 denotes the difference in outcomes between the SSP2-2.6 and SSP2-7.0 scenarios. Bars indicate the multi-model median, whereas symbols represent results from individual global agricultural economic models.

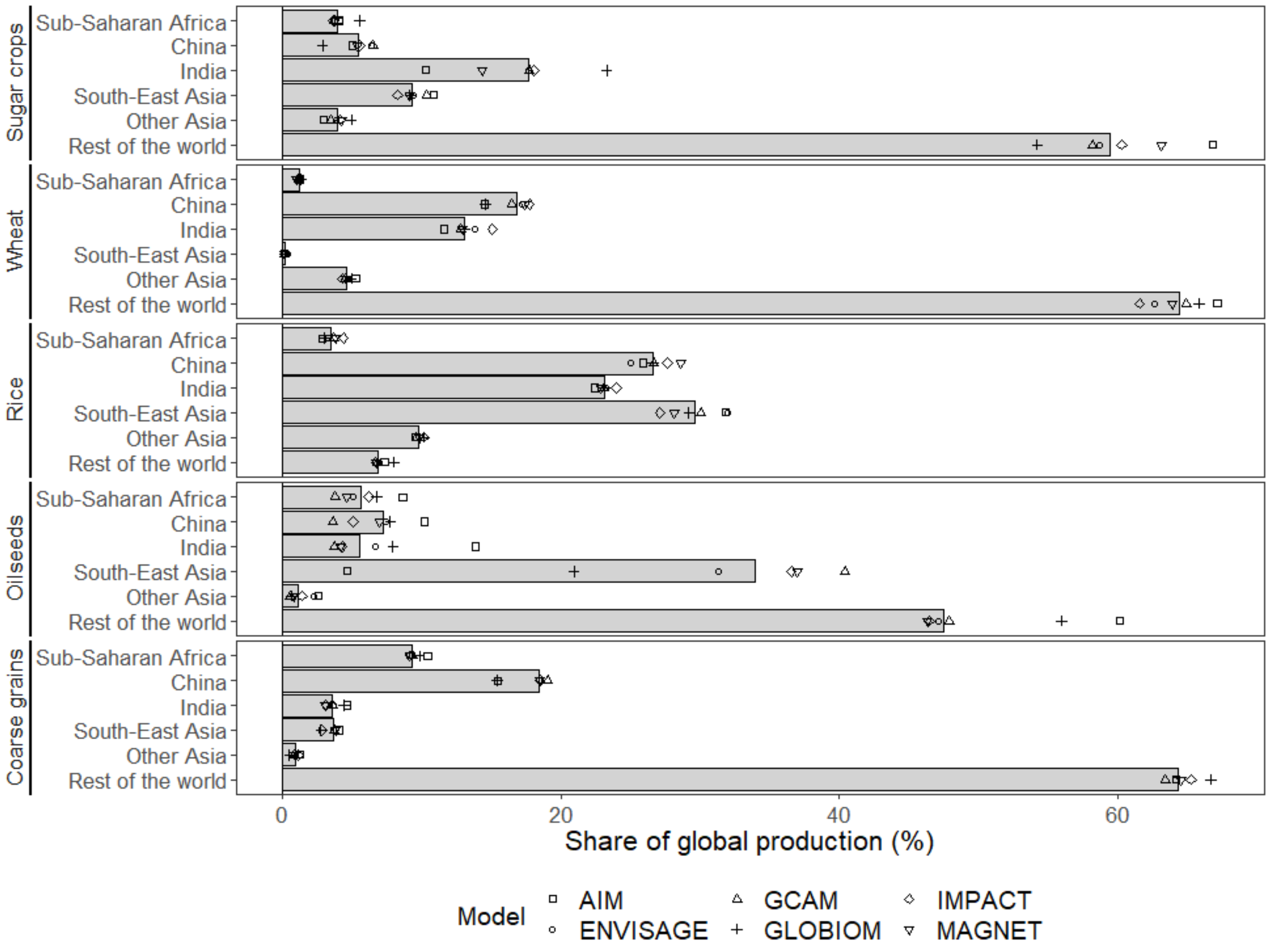

Supplementary Fig. 11. Share of the current global production volume (in tons) of major crops projected for 2050. Bars indicate the multi-model median, whereas symbols represent results from individual global agricultural economic models.

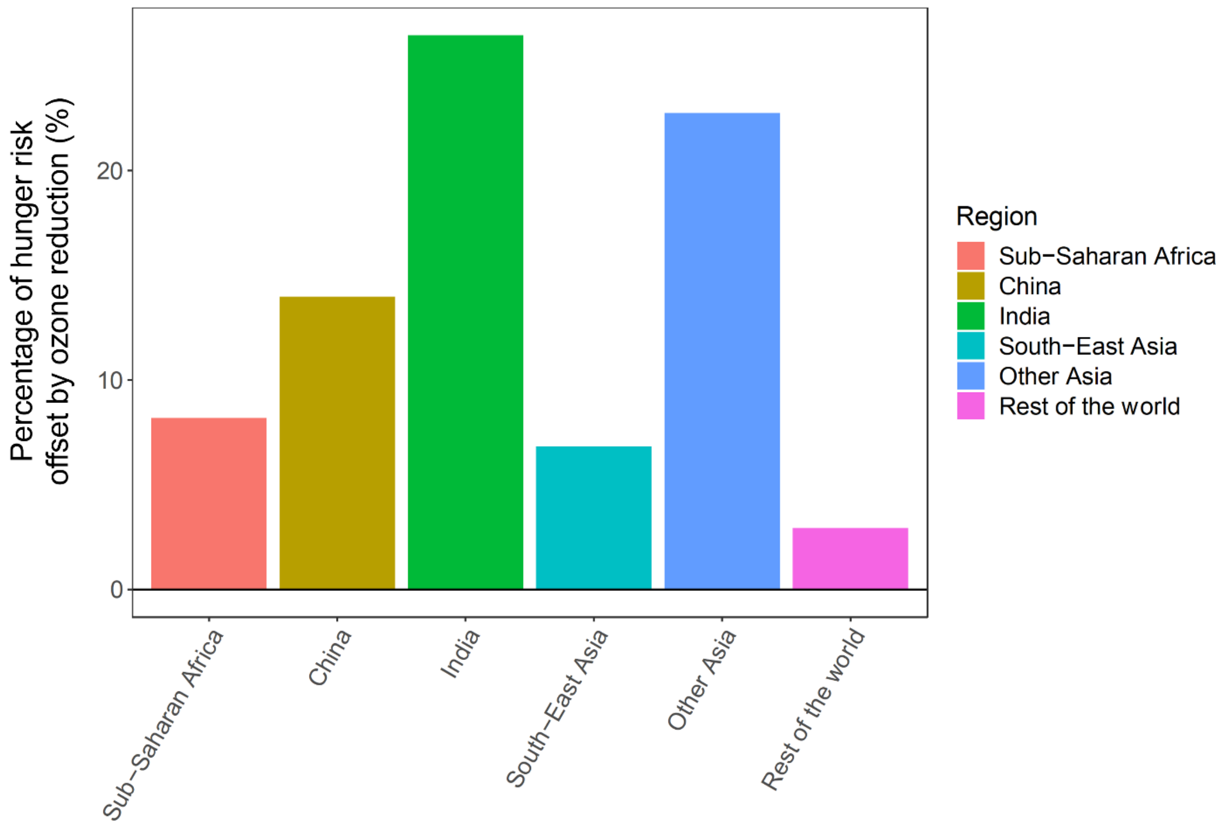

Supplementary Fig. 12. Percentage of mitigation-induced hunger risk offset by the accompanying ozone reduction in 2050. Bars indicate the multi-model median, based on results from six global agricultural economic models.

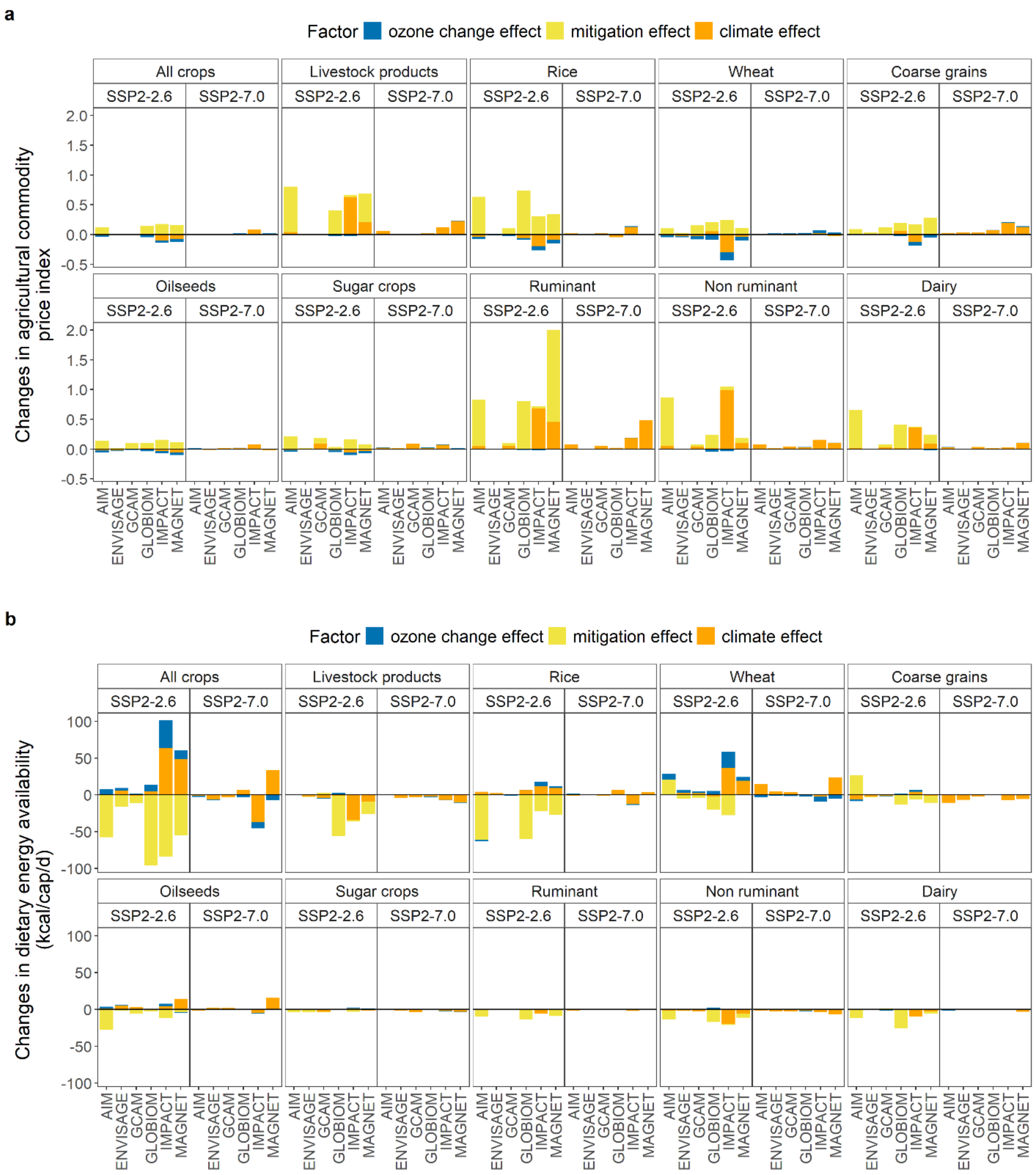

Supplementary Fig. 13. Changes in global mean agricultural commodity prices (a) and calorie availability (b) of each food commodity in 2050 from the baseline scenario.

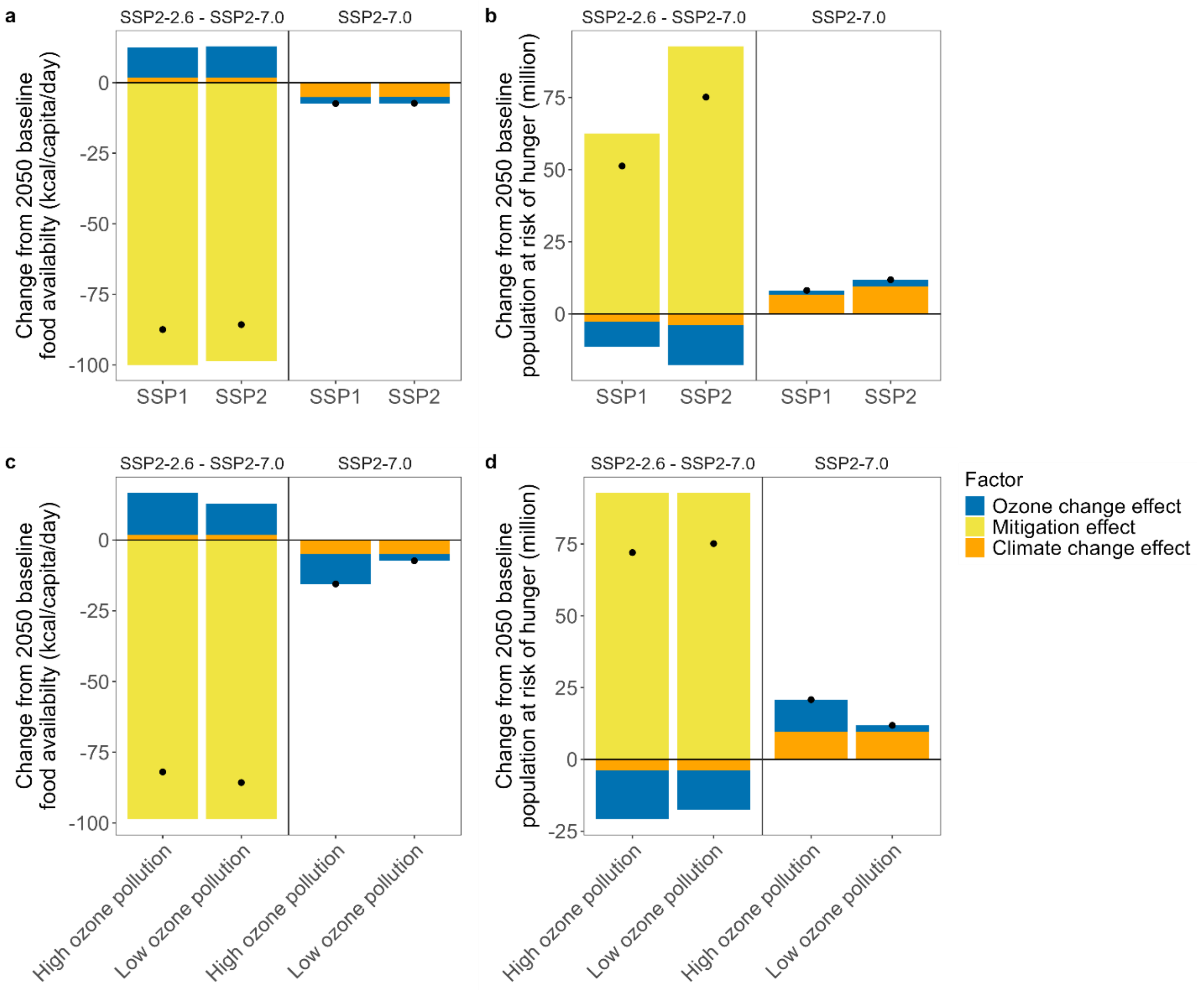

Supplementary Fig. 14. Socioeconomic and air quality uncertainties in food security. a-b, Projected food availability changes (a) and the population at risk of hunger (b) from the baseline scenario under SSP1 and SSP2 socioeconomic assumptions in 2050, resulting from each of the three factors. **c-d**, Changes in food security under higher ozone pollution levels compared to those (low ozone pollution in SSP2-2.6 and SSP2-7.0) used in our earlier results. Black dots indicate the combined effect of these three factors. The expression SSP2-2.6 – SSP2-7.0 denotes the difference in outcomes between the SSP2-2.6 and SSP2-7.0 scenarios.

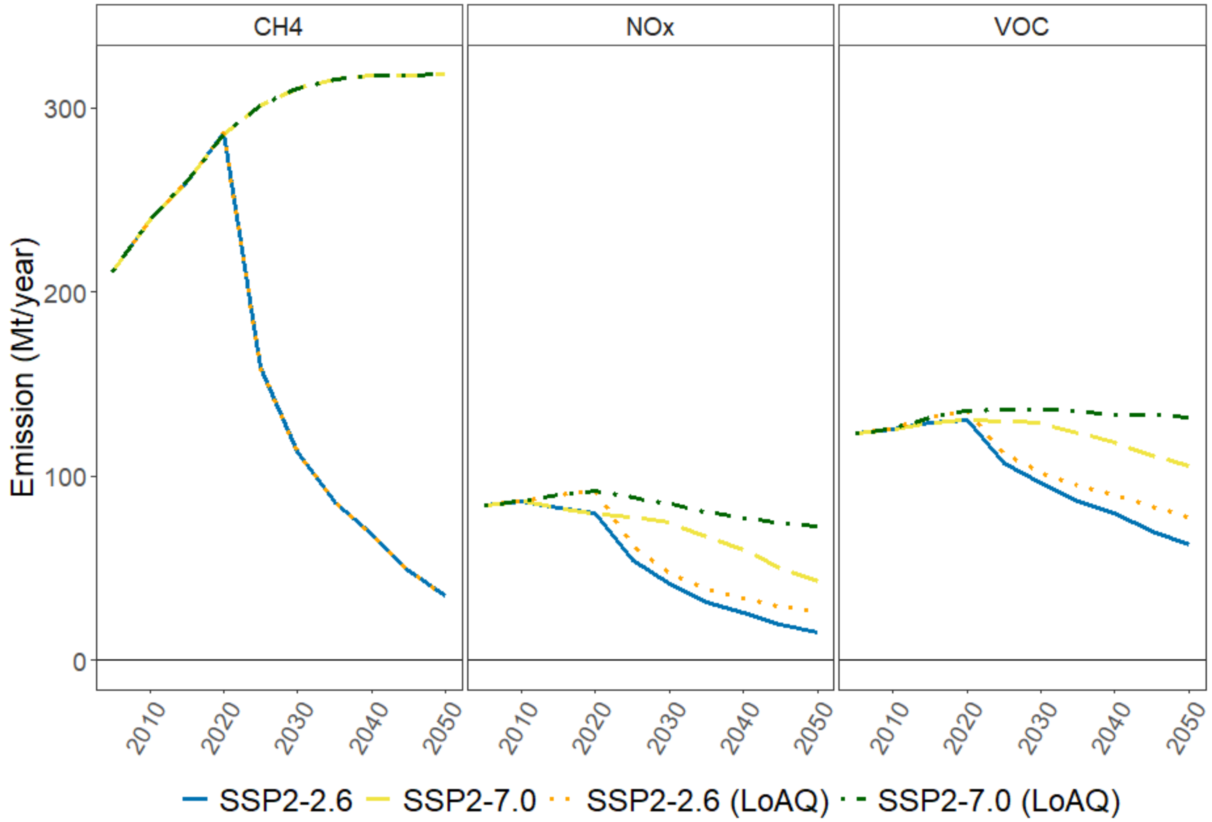

Supplementary Fig. 15. Changes in CH4, NOx and VOC emissions under the SSP2-2.6 and SSP2-7.0 scenarios over time, based on projections from the AIM/Hub model. LoAQ refers to the emissions under low air quality conditions.

2. Supplementary tables

Supplementary Table 1. Decomposition method.

Scenario \ Factor	Climate change effect	Mitigation effect	Ozone change effect
SSP2-2.6	BAU_MITI_RCP26 – BAU_MITI + BAU – BAU_NoCC	BAU_MITI – BAU	OZBAU_MITI_RCP26 – BAU_MITI_RCP26
SSP2-7.0	BAU – BAU_NoCC		OZBAU – BAU

Note: BAU_MITI and BAU scenarios were run under the SSP2-7.0 setting, but with a mitigation policy aligned with the 1.5°C target. All the scenarios are created to support the Second EAT-Lancet Commission report[3,4].

Supplementary Table 2. Exposure-response functions (ERFs) used to evaluate the relative yield loss.

Crop types	ERFs used for RYL calculation
Rice	0.00415×AOT40
Maize	0.00356×AOT40
Wheat	0.0163×AOT40
Oil crops	0.0113×AOT40
Sugar crops	0.0150×AOT40

AOT40 (Accumulated Exposure Over Threshold of 40 ppb) is the most commonly used metric for assessing O_3 exposure and its effects on vegetation. RYL: relative yield loss.

Supplementary Table 3. Regional categories used in the Agricultural Model Intercomparison and Improvement Project (AgMIP)[5].

Code	Region	Details	Notes
CAN	Canada	Canada	
USA	USA	United States of America	
BRA	Brazil	Brazil	
OSA	Other S&C America	Other South, Central America & Caribbean (incl. Mexico)	
FSU	Former Soviet Union	Former Soviet Union (European and Asian)	
EUR	Europe	Europe (excl. Turkey)	
MEN	Middle-East/North Afr.	Middle-East / North Africa (incl. Turkey)	
SSA	Sub-Saharan Afr.	Sub-Saharan Africa	
CHN	China	China (incl Hong-Kong, Macao)	
IND	India	India	
SEA	South-East Asia	South-East Asia (incl. Japan, Taiwan)	
OAS	Other Asia	Other Asia (incl. Other Oceania)	
ANZ	Australia/New Zealand	Australia/New Zealand	
NAM	North America	North America (Canada & USA)	CAN+USA
OAM	S&C America	Other Americas (South, Central & Caribbean)	BRA+OSA
AME	Africa & Middle East	Africa & Middle East	MEN + SSA
SAS	Southern Asia	Southern Asia	CHN + IND +SEA + OAS

Note: These regions are further aggregated into SSA, CHN, IND, SEA, OAS, and the Rest of the World in our analysis.

Supplementary Table 4. Regional mapping.

Country	ISO3	This study	Country	ISO3	This study
Afghanistan	AFG	OAS	Macao, China	MAC	CHN
Albania	ALB	EUR	Madagascar	MDG	SSA
Algeria	DZA	MEN	Malawi	MWI	SSA
Andorra	AND	EUR	Malaysia	MYS	SEA
Angola	AGO	SSA	Maldives	MDV	OAS
Anguilla	AIA	OSA	Mali	MLI	SSA
Antigua and Barbuda	ATG	OSA	Malta	MLT	EUR
Argentina	ARG	OSA	Marshall Islands	MHL	OAS
Armenia	ARM	FSU	Mauritania	MRT	SSA
Aruba	ABW	OSA	Mauritius	MUS	SSA
Australia	AUS	ANZ	Mexico	MEX	OSA
Austria	AUT	EUR	Micronesia (Federated States of)	FSM	OAS
Azerbaijan	AZE	FSU	Monaco	MCO	EUR
Bahamas	BHS	OSA	Mongolia	MNG	OAS
Bahrain	BHR	MEN	Montenegro	MNE	EUR
Bangladesh	BGD	OAS	Montserrat	MSR	OSA
Barbados	BRB	OSA	Morocco	MAR	MEN
Belarus	BLR	FSU	Mozambique	MOZ	SSA
Belgium	BEL	EUR	Myanmar (Burma)	MMR	SEA
Belize	BLZ	OSA	Namibia	NAM	SSA
Benin	BEN	SSA	Nauru	NRU	OAS
Bermuda	BMU	OSA	Nepal	NPL	OAS
Bhutan	BTN	OAS	Netherlands	NLD	EUR
Bolivia	BOL	OSA	Netherlands Antilles	ANT	OSA
Bosnia and Herzegovina	BIH	EUR	New Caledonia	NCL	OAS
Botswana	BWA	SSA	New Zealand	NZL	ANZ
Brazil	BRA	BRA	Nicaragua	NIC	OSA
British Virgin Islands	VGB	OSA	Niger	NER	SSA
Brunei Darussalam	BRN	SEA	Nigeria	NGA	SSA

Bulgaria	BGR	EUR	Niue	NIU	OAS
Burkina Faso	BFA	SSA	Norway	NOR	EUR
Burundi	BDI	SSA	Oman	OMN	MEN
Cambodia	KHM	SEA	Pakistan	PAK	OAS
Cameroon	CMR	SSA	Palau	PLW	OAS
Canada	CAN	CAN	Palestinian Territories	PSE	MEN
Cape Verde	CPV	SSA	Panama	PAN	OSA
Cayman Islands	CYM	OSA	Papua New Guinea	PNG	OAS
Cent African Rep	CAF	SSA	Paraguay	PRY	OSA
Chad	TCD	SSA	Peru	PER	OSA
Chile	CHL	OSA	Philippines	PHL	SEA
China	CHN	CHN	Poland	POL	EUR
Colombia	COL	OSA	Portugal	PRT	EUR
Comoros	COM	SSA	Qatar	QAT	MEN
Congo	COG	SSA	Rep. of Korea	KOR	SEA
Cook Islands	COK	OAS	Rep. of Moldova	MDA	FSU
Costa Rica	CRI	OSA	Reunion	REU	SSA
Côte d'Ivoire	CIV	SSA	Romania	ROU	EUR
Croatia	HRV	EUR	Russian Federation	RUS	FSU
Cuba	CUB	OSA	Rwanda	RWA	SSA
Cyprus	CYP	EUR	Saint Kitts and Nevis	KNA	OSA
Czech Republic	CZE	EUR	Saint Lucia	LCA	OSA
People's Republic of Korea	PRK	SEA	Saint Vincent and the Grenadines	VCT	OSA
Dem. Rep. of Congo	COD	SSA	Samoa	WSM	OAS
Denmark	DNK	EUR	San Marino	SMR	EUR
Djibouti	DJI	SSA	São Tomé and Principe	STP	SSA
Dominica	DMA	OSA	Saudi Arabia	SAU	MEN
Dominican Republic	DOM	OSA	Senegal	SEN	SSA
Ecuador	ECU	OSA	Serbia	SRB	EUR
Egypt	EGY	MEN	Seychelles	SYC	SSA
El Salvador	SLV	OSA	Sierra Leone	SLE	SSA
Equatorial Guinea	GNQ	SSA	Singapore	SGP	SEA
Eritrea	ERI	SSA	Slovakia	SVK	EUR

Estonia	EST	EUR
Ethiopia	ETH	SSA
Falkland Islands	FLK	OSA
Fiji	FJI	OAS
Finland	FIN	EUR
France	FRA	EUR
French Polynesia	PYF	OAS
Gabon	GAB	SSA
Gambia	GMB	SSA
Georgia	GEO	FSU
Germany	DEU	EUR
Ghana	GHA	SSA
Gibraltar	GIB	EUR
Greece	GRC	EUR
Grenada	GRD	OSA
Guadeloupe	GLP	OSA
Guatemala	GTM	OSA
Guinea	GIN	SSA
Guinea-Bissau	GNB	SSA
Guyana	GUY	OSA
Haiti	HTI	OSA
Holy See	VAT	EUR
Honduras	HND	OSA
Hong Kong	HKG	CHN
Hungary	HUN	EUR
Iceland	ISL	EUR
India	IND	IND
Indonesia	IDN	SEA
Iran	IRN	MEN
Iraq	IRQ	MEN
Ireland	IRL	EUR
Israel	ISR	MEN
Italy	ITA	EUR
Jamaica	JAM	OSA
Slovenia	SVN	EUR
Solomon Islands	SLB	OAS
Somalia	SOM	SSA
South Africa	ZAF	SSA
Spain	ESP	EUR
Sri Lanka	LKA	OAS
Sudan	SDN	SSA
Suriname	SUR	OSA
Swaziland	SWZ	SSA
Sweden	SWE	EUR
Switzerland	CHE	EUR
Syrian Arab Republic	SYR	MEN
Tajikistan	TJK	FSU
Thailand	THA	SEA
Macedonia	MKD	EUR
Timor-Leste	TLS	SEA
Togo	TGO	SSA
Tokelau	TKL	OAS
Tonga	TON	OAS
Trinidad and Tobago	TTO	OSA
Tunisia	TUN	MEN
Turkey	TUR	MEN
Turkmenistan	TKM	FSU
Turks and Caicos Islands	TCA	OSA
Tuvalu	TUV	OAS
Uganda	UGA	SSA
Ukraine	UKR	FSU
United Arab Emirates	ARE	MEN
United Kingdom	GBR	EUR
Tanzania	TZA	SSA
United States	USA	USA
Uruguay	URY	OSA
Uzbekistan	UZB	FSU
Vanuatu	VUT	OAS

Japan	JPN	SEA	Venezuela	VEN	OSA
Jordan	JOR	MEN	Vietnam	VNM	SEA
Kazakhstan	KAZ	FSU	Yemen	YEM	MEN
Kenya	KEN	SSA	Zambia	ZMB	SSA
Kiribati	KIR	OAS	Zimbabwe	ZWE	SSA
Kuwait	KWT	MEN	French Guiana	GUF	OSA
Kyrgyzstan	KGZ	FSU	Western Sahara	ESH	MEN
Lao People's Dem. Rep.	LAO	SEA	Taiwan	TWN	SEA
Latvia	LVA	EUR	Greenland	GRL	EUR
Lebanon	LBN	MEN	Svalbard and Jan Mayen	SJM	EUR
Lesotho	LSO	SSA	Puerto Rico	PRI	OSA
Liberia	LBR	SSA	Isle of Man	IMN	EUR
Libyan Arab Jamahiriya	LBY	MEN	Jersey	JEY	EUR
Liechtenstein	LIE	EUR	Guernsey	GGY	EUR
Lithuania	LTU	EUR	US Virgin Islands	VIR	OSA
Luxembourg	LUX	EUR			

Reference

1. SSP Database (Shared Socioeconomic Pathways) - Version 2.0 (IIASA, 2018); https://tntcat.iiasa.ac.at/SspDb

2. Fricke, O. et al. The marker quantification of the Shared Socioeconomic Pathway 2: A middle-of-the-road scenario for the 21st century. *Glob. Environ. Change* **42**, 251 (2017).

3. Sundiang, M. et al. Bundling measures for food systems transformation: a global, multimodel assessment. *Lancet Planet. Health* **9**, 101339 (2025).

4. Rockström, J., S. et al. EAT-Lancet Commission on healthy, sustainable, and just food systems. *Lancet* **406**, 1625–1700 (2025).

5. Rosenzweig, C. et al. The agricultural model intercomparison and improvement project (AgMIP): protocols and pilot studies. *Agric. For. Meteorol.* **170**, 166–182 (2013).
