# Supplementary material for: Ozone pollution reduction partially offsets the negative impact of climate change mitigation efforts on global hunger
Source: Nat Food. 2026 Mar 16;7(4):356–68. doi: 10.1038/s43016-026-01322-3 (PMC13121001; doi:10.1038/s43016-026-01322-3)
Supplement: Supplementary file 2 — Reporting Summary [file 43016_2026_1322_MOESM2_ESM.pdf]

nature portfolio

Corresponding author(s): Shujuan Xia, Tomoko Hasegawa, Shinichiro Fujimori

Last updated by author(s): Jan 29, 2026

Reporting Summary

Nature Portfolio wishes to improve the reproducibility of the work that we publish. This form provides structure for consistency and transparency in reporting. For further information on Nature Portfolio policies, see our Editorial Policies and the Editorial Policy Checklist.

Statistics

For all statistical analyses, confirm that the following items are present in the figure legend, table legend, main text, or Methods section.

n/a	Confirmed	
☐	☒	The exact sample size (*n*) for each experimental group/condition, given as a discrete number and unit of measurement
☒	☐	A statement on whether measurements were taken from distinct samples or whether the same sample was measured repeatedly
☒	☐	The statistical test(s) used AND whether they are one- or two-sided<br>*Only common tests should be described solely by name; describe more complex techniques in the Methods section.*
☒	☐	A description of all covariates tested
☐	☒	A description of any assumptions or corrections, such as tests of normality and adjustment for multiple comparisons
☐	☒	A full description of the statistical parameters including central tendency (e.g. means) or other basic estimates (e.g. regression coefficient) AND variation (e.g. standard deviation) or associated estimates of uncertainty (e.g. confidence intervals)
☒	☐	For null hypothesis testing, the test statistic (e.g. *F*, *t*, *r*) with confidence intervals, effect sizes, degrees of freedom and *P* value noted<br>*Give P values as exact values whenever suitable.*
☒	☐	For Bayesian analysis, information on the choice of priors and Markov chain Monte Carlo settings
☒	☐	For hierarchical and complex designs, identification of the appropriate level for tests and full reporting of outcomes
☒	☐	Estimates of effect sizes (e.g. Cohen's *d*, Pearson's *r*), indicating how they were calculated

Our web collection on statistics for biologists contains articles on many of the points above.

Software and code

Policy information about availability of computer code

Data collection	GAMS and R
Data analysis	All figures were generated using R (version 4.5.0), with key packages including dplyr (version 1.1.4), ggplot2 (version 3.5.2), and tidyverse (version 2.0.0).

For manuscripts utilizing custom algorithms or software that are central to the research but not yet described in published literature, software must be made available to editors and reviewers. We strongly encourage code deposition in a community repository (e.g. GitHub). See the Nature Portfolio guidelines for submitting code & software for further information.

Data

Policy information about availability of data

All manuscripts must include a data availability statement. This statement should provide the following information, where applicable:

- Accession codes, unique identifiers, or web links for publicly available datasets
- A description of any restrictions on data availability
- For clinical datasets or third party data, please ensure that the statement adheres to our policy

The data used to generate these figures are available at https://figshare.com/s/3d7dcfb24ae66de3d35c

Research involving human participants, their data, or biological material

Policy information about studies with human participants or human data. See also policy information about sex, gender (identity/presentation), and sexual orientation and race, ethnicity and racism.

Reporting on sex and gender	n/a
Reporting on race, ethnicity, or other socially relevant groupings	n/a
Population characteristics	n/a
Recruitment	n/a
Ethics oversight	n/a

Note that full information on the approval of the study protocol must also be provided in the manuscript.

Field-specific reporting

Please select the one below that is the best fit for your research. If you are not sure, read the appropriate sections before making your selection.

☐ Life sciences ☐ Behavioural & social sciences ☒ Ecological, evolutionary & environmental sciences

For a reference copy of the document with all sections, see nature.com/documents/nr-reporting-summary-flat.pdf

Ecological, evolutionary & environmental sciences study design

All studies must disclose on these points even when the disclosure is negative.

Study description	This study evaluates the trade-offs and co-benefits of climate mitigation policies on global food security by using six integrated assessment models (IAMs). Specifically, it assesses how the reduction in co-emitted air pollutants under climate mitigation scenarios affects surface ozone levels, and how those ozone reductions subsequently impact agricultural yields and global hunger outcomes.
Research sample	It comprises outputs from six global IAMs that simulate agricultural and economic responses to a range of climate and policy scenarios. These models cover multiple world regions, staple crops, and population segments, allowing for a detailed assessment of hunger risks and agricultural productivity through 2050.
Sampling strategy	The study uses a scenario-based sampling strategy. Six policy scenarios (baseline and mitigation pathways consistent with Shared Socioeconomic Pathways and Representative Concentration Pathways) were applied across all six IAMs.
Data collection	Data are collected from each modeling team participating in the intercomparison.
Timing and spatial scale	Simulations project impacts through the year 2050, with outputs typically provided in five-year intervals. The analysis is conducted at a global scale, with results disaggregated to regional levels.
Data exclusions	No data are excluded.
Reproducibility	The overall framework, scenario setup, and analysis methods are documented and can be reproduced.
Randomization	No randomization.
Blinding	No blinding.

Did the study involve field work? ☐ Yes ☒ No

Reporting for specific materials, systems and methods

We require information from authors about some types of materials, experimental systems and methods used in many studies. Here, indicate whether each material, system or method listed is relevant to your study. If you are not sure if a list item applies to your research, read the appropriate section before selecting a response.

Materials & experimental systems

n/a	Involved in the study
☒	☐ Antibodies
☒	☐ Eukaryotic cell lines
☒	☐ Palaeontology and archaeology
☒	☐ Animals and other organisms
☒	☐ Clinical data
☒	☐ Dual use research of concern
☒	☐ Plants

Methods

n/a	Involved in the study
☒	☐ ChIP-seq
☒	☐ Flow cytometry
☒	☐ MRI-based neuroimaging

Plants

Seed stocks	n/a
Novel plant genotypes	n/a
Authentication	n/a
